# The PCNA interaction motifs revisited: thinking outside the PIP-box

**DOI:** 10.1007/s00018-019-03150-0

**Published:** 2019-05-27

**Authors:** Andreas Prestel, Nanna Wichmann, Joao M. Martins, Riccardo Marabini, Noah Kassem, Sebastian S. Broendum, Marit Otterlei, Olaf Nielsen, Martin Willemoës, Michael Ploug, Wouter Boomsma, Birthe B. Kragelund

**Affiliations:** 1 Department of Biology, University of Copenhagen, Ole Maaloes Vej 5, 2200 Copenhagen N, Denmark; 2 Department of Computer Science, University of Copenhagen, Universitetsparken 1, 2100 Copenhagen Ø, Denmark; 3 Present Address: Department of Biochemistry and Molecular Biology, Biomedicine Discovery Institute, Monash University, Victoria, 3800 Australia; 4 Department of Clinical and Molecular Medicine, Faculty of Medicine and Health Sciences, NTNU Norwegian University of Science and Technology, 7491 Trondheim, Norway; 5 Finsen Laboratory, Rigshospitalet, Ole Maaloes Vej 5, 2200 Copenhagen N, Denmark; 6 Finsen Laboratory, Biotechnology Research Innovation Centre, University of Copenhagen, Ole Maaloes Vej 5, 2200 Copenhagen N, Denmark

**Keywords:** APIM, DNA homeostasis, IDP, Intrinsically disordered, NMR, SLiM, p21

## Abstract

Proliferating cell nuclear antigen (PCNA) is a cellular hub in DNA metabolism and a potential drug target. Its binding partners carry a short linear motif (SLiM) known as the PCNA-interacting protein-box (PIP-box), but sequence-divergent motifs have been reported to bind to the same binding pocket. To investigate how PCNA accommodates motif diversity, we assembled a set of 77 experimentally confirmed PCNA-binding proteins and analyzed features underlying their binding affinity. Combining NMR spectroscopy, affinity measurements and computational analyses, we corroborate that most PCNA-binding motifs reside in intrinsically disordered regions, that structure preformation is unrelated to affinity, and that the sequence-patterns that encode binding affinity extend substantially beyond the boundaries of the PIP-box. Our systematic multidisciplinary approach expands current views on PCNA interactions and reveals that the PIP-box affinity can be modulated over four orders of magnitude by positive charges in the flanking regions. Including the flanking regions as part of the motif is expected to have broad implications, particularly for interpretation of disease-causing mutations and drug-design, targeting DNA-replication and -repair.

**Electronic supplementary material:**

The online version of this article (10.1007/s00018-019-03150-0) contains supplementary material, which is available to authorized users.

## Introduction

Protein–protein interactions are essential for all biological processes, especially cellular regulation and signaling. These processes often involve pathways where the ability to associate with multiple targets is important. Compared to folded proteins, intrinsically disordered proteins (IDPs) have large accessible surface areas which increase their potential to interact with multiple binding partners through short linear motifs (SLiMs) [1–3]. Intriguingly, the human proteome has been estimated to contain more than a hundred thousand—possibly up to a million—different SLiMs, most of which remain to be discovered and understood [4]. This knowledge void limits our understanding of many important biological processes and is rooted in the low sequence conservation of IDPs [5], the existence of only a few core positions of importance in the motif [6, 7], and their experimentally challenging discovery path [8, 9].

**Fig. 1:**
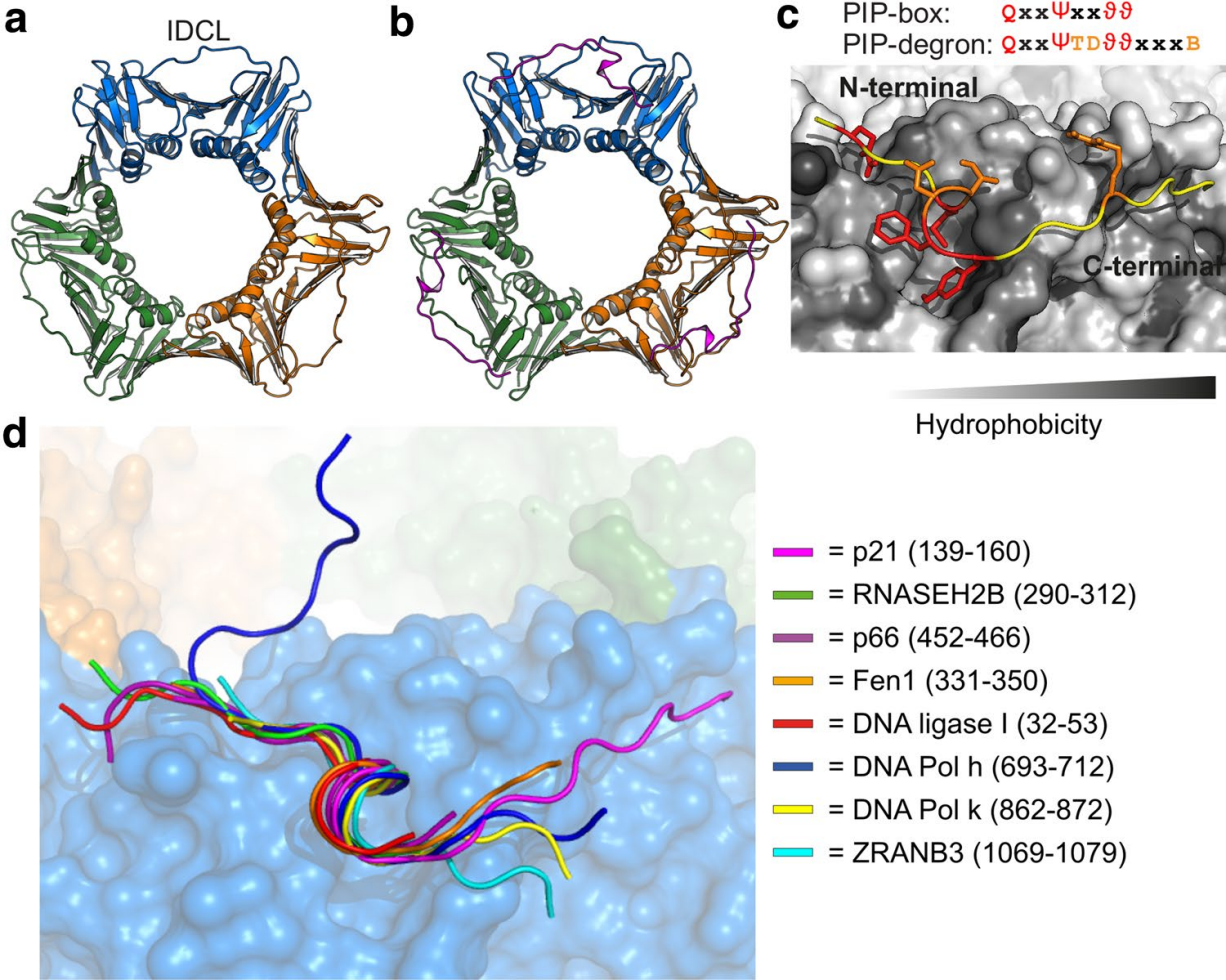
PCNA is a circular trimer that binds disordered ligands through short linear motifs. **a** Structure of unbound human PCNA (PDB-code:1VYM) highlighting the three different subunits colored in blue, orange and green, respectively, and the interdomain connecting loop (IDCL) indicated. **b** Structure of p21 (magenta) bound to PCNA (PDB-code: 1AXC; coloring as in **a**). **c** Magnification of the binding pockets of PCNA with bound p21. The binding pocket is made from residues 40–44, 117–135 (IDCL), 230–235, and 251–253. The PCNA surface is colored in gray shades according to hydrophobicity; the PIP-box residues inserted into the binding pockets are highlighted in red and degron specific residues in orange. **d** Overlay of seven peptides crystallized in complex with human PCNA including a degron (p21) and an APIM (ZRANB3). The PCNA surface is from the p21-complex (PDB-code: 1AXC) and colored as described in **a**

Proliferating cell nuclear antigen (PCNA) is a cellular hub protein located at the heart of a complex protein network mediating DNA replication and repair, chromatin remodeling, and epigenetics through interactions facilitated by SLiMs [10]. It is loaded onto DNA by the replication factor C complex [11, 12] and functions as a cyclic homotrimer. Each subunit consists of two homologous domains that are connected by the interdomain connecting loop (IDCL), Fig. 1a, which together with underlying hydrophobic pockets, constitute three identical binding sites for a large set of diverse ligands, Fig. 1b–d. Through binding of these, PCNA fulfills many functions. It acts as a processivity factor for DNA polymerases, tethering them to the DNA, thus increasing their processivity rates from tens to thousands of nucleotides per second [13–15]. When loaded onto DNA, PCNA enforces replication and repair by recruitment of specialized polymerases (pol) such as pol δ during replication [16], and pol η upon DNA damage [17]. PCNA is also involved in orchestrating other replication events [18], chromatin assembly [19], and preventing re-replication of DNA (reviewed in [10]) involving ligands such as p21 [20], p53 [21], and p300 [22]. Importantly, PCNA also participates in protein degradation via presentation of ligands to the CRL4^Cdt2^ ubiquitylation complex [23, 24] leading to their proteasomal degradation. Thus, PCNA is a folded cellular hub with a huge and diverse interactome.

Many PCNA-interacting proteins (PIPs) have a characteristic SLiM called a PIP-box [25], or, for degradation, a PIP-degron [26], Fig. 1c. The canonical PIP-box motif is QxxΨxxϑϑ, where Ψ is an aliphatic hydrophobic residue (L, M, I ,V), ϑ is aromatic (most often Y or F), and x can be any amino acid. The canonical PIP-degron extends the PIP-box. It harbors a basic residue (K, R) in the + 4 position from the last aromatic residue of the PIP-box [26], Fig. 1c, as well as a threonine and an aspartic acid (TD) just before the aromatics, which strengthened binding to PCNA [26, 27]. By itself the TD do not lead to degradation, as FEN1 mutants harboring the TD motif in the PIP-box, but lacking the basic residue in the + 4 position have been shown to be stable [20, 26, 27].

In addition to the apparent differentiation between PIP-boxes and -degrons, there are other, more divergent sequences binding to PCNA. Within recent years, new and alternative PCNA binding motifs have been described, including the AlkB homologue 2 PCNA-interacting motif (APIM) with the five consensus residues [K/R]–[F/Y/W]–[L/I/V/A]–[L/I/V/A]–[K/R], also identified in various proteins in the cytosol [28, 29]. Moreover, it was recently proposed that many PIP-boxes and -degrons contain overlapping motifs that obscure the motif fingerprint, including the Rev1-interacting region (RIR)- and the Mlh-1 interacting proteins (MIP) motifs [30].

Structural studies of PIP-box- and -degron-containing proteins and/or their derived peptides in complex with PCNA have revealed how the PIP-box motif mediates PCNA interaction. The crystal structure of p21 bound to human PCNA [20] was the first to provide molecular insight, and structures of several PCNA-complexes with different PIP-box peptides and PCNA from six different species have since been solved [20, 31–45]. Here, all PIP-box and PIP-degron-containing peptides adopt essentially the same conformation when bound (Fig. 1b, d): an extended N-terminal region, a single, four-residue α- or 3_10_-helix turn encompassing the hydrophobic residues of the PIP-box, and an extended region C-terminal to the PIP-box of variable length, sometimes forming a β-strand with the IDCL [20]. The turn structure positions the three conserved hydrophobic and aromatic residues of the PIP-motifs into the hydrophobic pockets of PCNA, whereas the glutamine inserts into the Q-pocket forming hydrogen bonds to backbone atoms of PCNA [20, 31, 39, 46]. The overall structure of PCNA is preserved in the bound form arguing against any gross ligand-induced conformational changes [20, 31, 39, 46].

Although the resemblance to the PIP-box and -degron is overall low, recent crystal structures of PCNA in complex with APIM peptides confirmed the exploitation of the same binding site and a similar binding mode [47, 48]. Thus, the PCNA binding pockets may be adaptable to a larger group of ligands than currently appreciated. It also suggests that there may be additional features extractable from this functional and sequential variation, which may be operational in determining PCNA selectivity and the affinity of the ligands—qualities that are essential to a cellular hub.

Given the critical processes controlled by PCNA and the intriguing sequence diversity, we were prompted to revisit the reported PIP-motifs in a quantitative and systematic study to clarify and delineate determinants and features that underlie motif interactions including potential non-SLiM encoded properties. From the literature, we have analyzed 77 PCNA ligands with a total of 83 confirmed PCNA interaction sites that are reported to bind to the same binding pockets on PCNA. We first show the motifs to reside predominantly in intrinsically disordered regions. Using NMR spectroscopy, we then demonstrate that in contrast to previous studies [49], preformation of structure in the ligands is uncorrelated to binding affinity. By combining a systematic review of current literature, various computational procedures, and experimental characterizations of binding affinities under identical conditions, we then show that the binding pockets of PCNA are more promiscuous than anticipated and that the electrostatic properties of the regions flanking the PCNA binding motifs, as suggested earlier [31], are so essential that they can modulate the affinity by four orders of magnitude. Our findings lay the foundation for a reassessment of this diverse and multifunctional class of short linear motifs, which may have consequences for future design of regulatory drugs targeting DNA replication and repair and for interpreting disease-related mutations in PCNA ligands.

## Results

To investigate the primary determinants underlying the affinity between PCNA and its binding partners, we started by considering the effect of secondary structure within the motif region, focusing on the degree of structure preformation and the degree of disorder over the entire length of the motif. We proceeded by analyzing the sequence variation in the motif region based on a curated set of experimentally confirmed binding partners. As a complementary perspective on the sequence preferences in the motif, we then characterized the amino acid preferences using a structure-based computational procedure. Finally, we experimentally probed the effect of charge in the flanking regions surrounding the motif.

### Preformed structure of PCNA motifs is unrelated to affinity

**Fig. 2:**
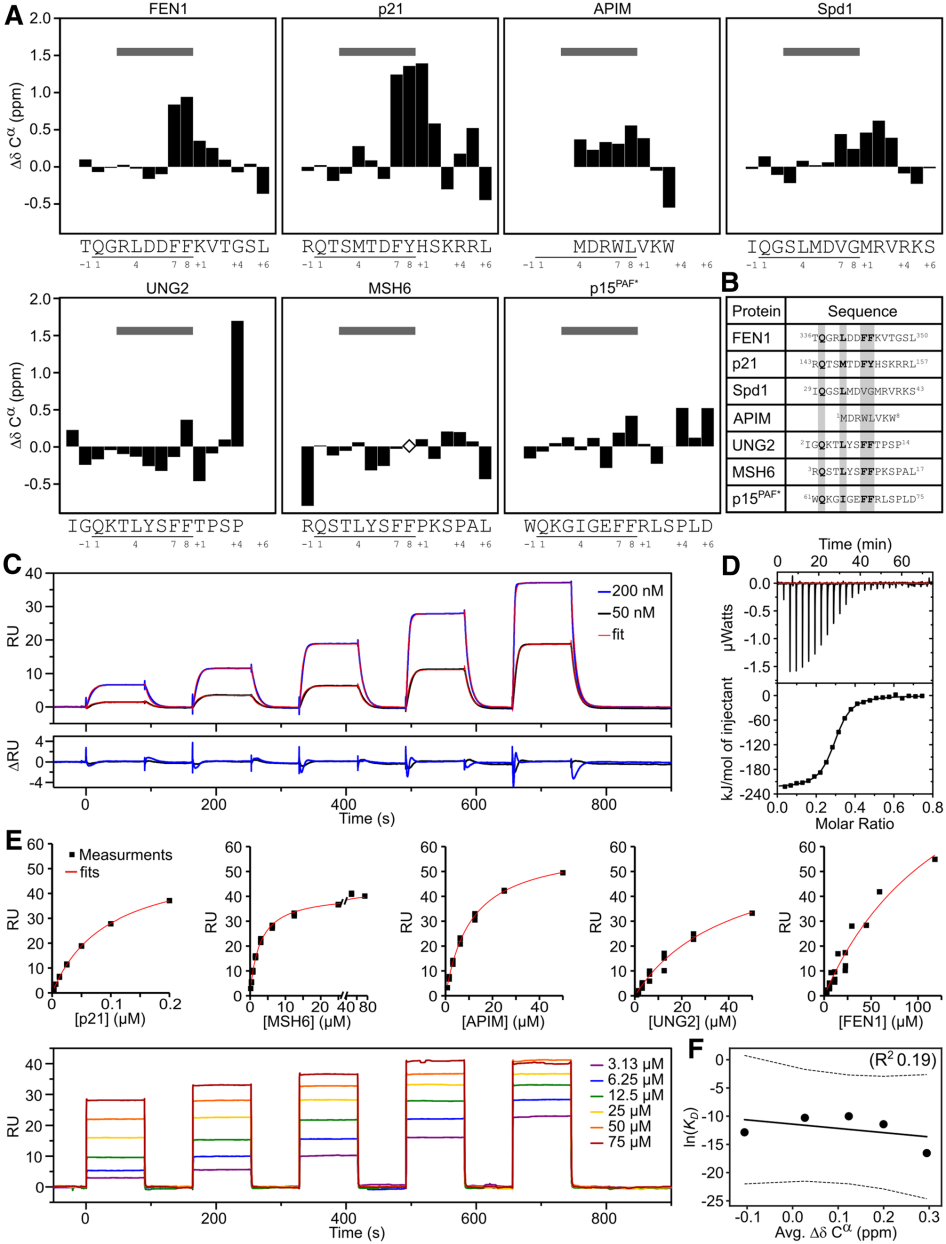
Preformed structure in disordered PCNA ligand motifs does not correlate to affinity. **a** Secondary C^α^-chemical shifts (ΔδC^α^) per residue of unbound PCNA target peptides. The sequences of the peptides and motif numbering (from − 2 to + 6) are shown below each plot, with PIP-motif-positions underlined. The positions of the 3_10_-helix-forming residues of p21 in complex with PCNA (position 3–8) are marked at the top with a gray bar. Open diamonds indicate lack of assignment. For Spd1 and p15^PAF^ the assignments are from full-length proteins. The MSH6, FEN1, p21, and APIM peptides were N-terminally acetylated and C-terminally amidated. *From BMRB entry 1933 [50]. **b** Alignment of peptide sequences with numbering from the full-length proteins. PIP box specific residues are highlighted in gray. **c** Binding of p21 to human PCNA by SPR. Sensorgrams were obtained by injecting a series of p21 concentrations over PCNA captured by the immobilized Anti-His_6_ antibody. The p21 concentrations were five serial twofold dilutions, injected in the order of increasing concentrations, with a final concentration of 50 nM (black) or 200 nM (blue). Non-linear regression fits are shown in red with residuals below. **d** Binding of p21 to human PCNA by ITC. In the representative ITC experiment PCNA was injected into the p21 peptide. The upper portion shows baseline-corrected raw data from the titration, and the low portion shows the normalized integrated binding isotherms with the fitted binding curves assuming a single set of equivalent binding sites. **e** Equilibrium SPR binding analyses of PCNA ligands (p21, MSH6, APIM, UNG2, and FEN1). The lower part shows a representative binding series of MSH6 to PCNA, with the concentration of the final injection indicated. The sensorgrams were obtained by injecting the respective peptide over PCNA captured by the immobilized anti-His_6_ antibody. The BIAevaluation software was used to extract the *K*_D_s, fits shown in red. **f** Correlation between average SCS of the PCNA ligands in **a** and ln(*K*_D_) (*K*_D_s in M as measured by SPR in **c** and **e**)

Several studies have investigated the structure propensity of PCNA ligands in the free state and while some found a significant amount of preformed helical structure [50, 51], a recent study with a locked PIP motif suggested that this does not correlate with affinity for p21 [52]. To elaborate on these findings, we selected a range of peptides representing variations of the PCNA binding motifs including the canonical PIP-box (FEN1: TQGRLDDFFKVTGSL, MSH6: RQSTLYSFFPKSPAL, and an UNG2 variant: MIGQKTLYSFFTPSP), PIP-degron (p21: RQTSMTDFYHSKRRL), and APIM (MDRWLVKW). In addition, a highly degenerate PIP-degron from Spd1 (IQGSLMDVGMRVRKS) was included in the analysis [53–55] as well as p15(PAF), for which assignments were already available [50]. Using heteronuclear NMR spectroscopy, ^13^C chemical shifts were assigned for all seven peptides and their secondary chemical shifts extracted using a reference coil chemical shift set for IDPs [56, 57], reporting on backbone dihedral preferences, Fig. 2a. To enable comparison of the motifs we used the following nomenclature in accordance with previous literature: The glutamine of the canonical PIP-box and -degron were defined as position 1, and the two aromatic residues as positions 7 and 8. Hence, residues N-terminal to the glutamine were designated a negative sign and residues C-terminal to position 8 a positive sign. APIM and PIP motifs were aligned based on a comparison of their orientation in the crystal structures (5MLW and 1AXC, respectively) and the core APIM motif [K/R]–[F/Y/W]–[L/I/V/A]–[L/I/V/A]–[K/R] was defined as position 6 to + 2. The calculated secondary chemical shifts of the selected set of peptides were then plotted against the sequence position, Fig. 2a.

The peptides were mainly disordered with little helical propensity. The largest helix propensities were seen for the peptides of p21 and FEN1 (C^α^ secondary chemical shifts above 0.1 ppm for residues at position 7 to + 2), for Spd1 (at positions 3 to + 4), and for the APIM peptide (at positions 4 to + 1), Fig. 2a. However, residues with the largest helical propensity did not match those forming the helix turn structure in the PCNA-bound complexes, Fig. 2a, gray bars. Furthermore, helix propensity was entirely absent in MSH6, UNG2, and p15(PAF).

To determine the binding affinities of the peptides for PCNA, we employed surface plasmon resonance (SPR) using a His_6_-tagged PCNA that was captured via surface linked anti-His_6_-antibodies. Individual kinetic rate constants could be determined only in the case of p21, which had a *k*_on_ of 1.7 ± 0.01 × 10^6^ s^−1^ M^−1^, and a *k*_off_ of 0.14 ± 0.0003 s^−1^, resulting in a *K*_D_ of 80 ± 0.3 nM, Fig. 2c. This is similar to previous reports [31, 46, 58] and was further corroborated using isothermal titration calorimetry (ITC), which gave a comparable affinity (*K*_D_ of 67 ± 9 nM), Fig. 2d. In both cases, we found a stoichiometry of three ligands bound to each PCNA trimer. Binding was enthalpy driven (Δ*H* = − 230 ± 17 kJ mol^−1^) with an entropic penalty (− *T*Δ*S* = 190 ± 17 kJ mol^−1^). For all other ligands, binding affinities were determined by SPR using the steady-state response at equilibrium (Fig. 2e, and Fig. S1, and Table 1). Despite having canonical PIP motifs, MSH6 (*K*_D_ = 3.0 ± 0.05 μM), UNG2 (*K*_D_ = 34 ± 5 μM) and FEN1 (*K*_D_ = 45 ± 17 μM) bind PCNA with 40–600 times lower affinity compared to p21. In the case of MSH6 and UNG2, the tenfold difference in affinity was also unexpected since the motifs are differing in only one residue (QSTLYSFF and QKTLYSFF, respectively). Since this residue is solvent exposed, it can point towards a role for features not linked to the motif itself. The APIM peptide differs from the others, both in length and sequence homology, but the affinity is in a similar range (*K*_D_ = 11 ± 0.5 μM). To analyze the effect of preformed structure in the peptides on the binding affinities, we calculated the average helicity over the motif and correlated this to affinity, Fig. 2f. As apparent, no significant correlation (*R*^2^ = 0.19) between these entities could be seen, suggesting that preformed structure is not a defining feature for high affinity.

**Table 1:** Affinities and thermodynamics of PCNA ligand binding

	*K*_D_ (M)	< Δ*δ* > *C*^α^ (ppm)/%helicity*, NMR	*K*_D_ (M)	Δ*H* (kJ mol^−1^)	− *T*Δ*S* (kJ mol^−1^)	Δ*G* (kJ mol^−1^)
	SPR		ITC	ITC	ITC	ITC
p21	8.0E − 08 ± 3.4E − 10	0.29/9%	6.7E − 08 ± 9.0E − 9	− 230 ± 17	− 190 ± 17	− 40 ± 17
MSH6	2.9E − 06 ± 2.0E − 07	− 0.11/0%				
APIM	1.1E − 05 ± 5.2E − 07	0.20/6.3%				
UNG2	3.4E − 05 ± 4.2E − 06	0.03/1%				
FEN1	4.5E − 05 ± 7.3E − 06	0.12/3.8%				

*Using 100% helicity as corresponding to a < Δ*δ* > *C*^α^ of 3.2 ppm

### A curated set of 77 PCNA interaction partners across species

As was evident from the above analysis, highly non-canonical motifs such as the APIM still bind PCNA with decent affinities. Recent work has shown PCNA motifs to be embedded within overlapping motifs [30], which forces the motif region to adopt sequence-patterns that deviate from canonicity. To scrutinize the effect of sequence variation in the PCNA-interacting motifs, we extended on previous work by Moldovan et al. [25], and assembled a large set of interaction partners confirmed to interact with PCNA via a SLiM (Table 2). In an attempt to eliminate false positives, our list is intentionally conservative, and likely not exhaustive, and only includes partners for which direct evidence of binding via a PIP motif or an APIM exists. A binding partner is included in the table if the interaction of the motif with PCNA has been confirmed either through a three-dimensional structure of the complex, by mutagenesis of specific SLiM-residues, or deletion of up to 50 residues containing the SLiM, leading to a decrease of affinity for PCNA. It is also included if binding or functional studies using peptides (restricted to a maximum length of 50 residues) confirmed an interaction. We included three designed, non-natural peptides (p21-like, [59] and APIM- and UNG variants, Table 2) and collated a total of 77 different ligands, of which nine were confirmed degrons, Table S1. The list harbors 83 different motifs representing seven species and all known classes of PCNA-mediated functionalities [25]: DNA replication, cell cycle control and survival, chromatin assembly and maintenance, sister chromatid cohesion, DNA repair, and DNA damage avoidance, Table 2.

**Table 2:**
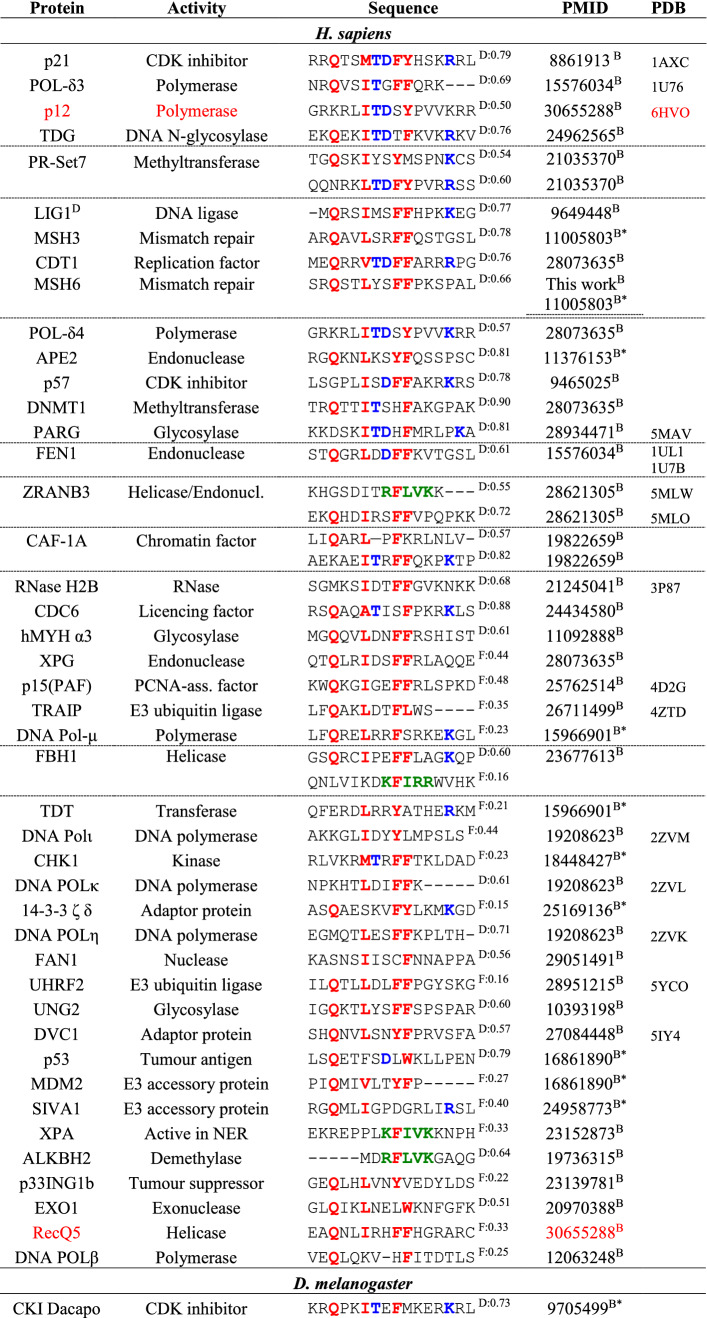

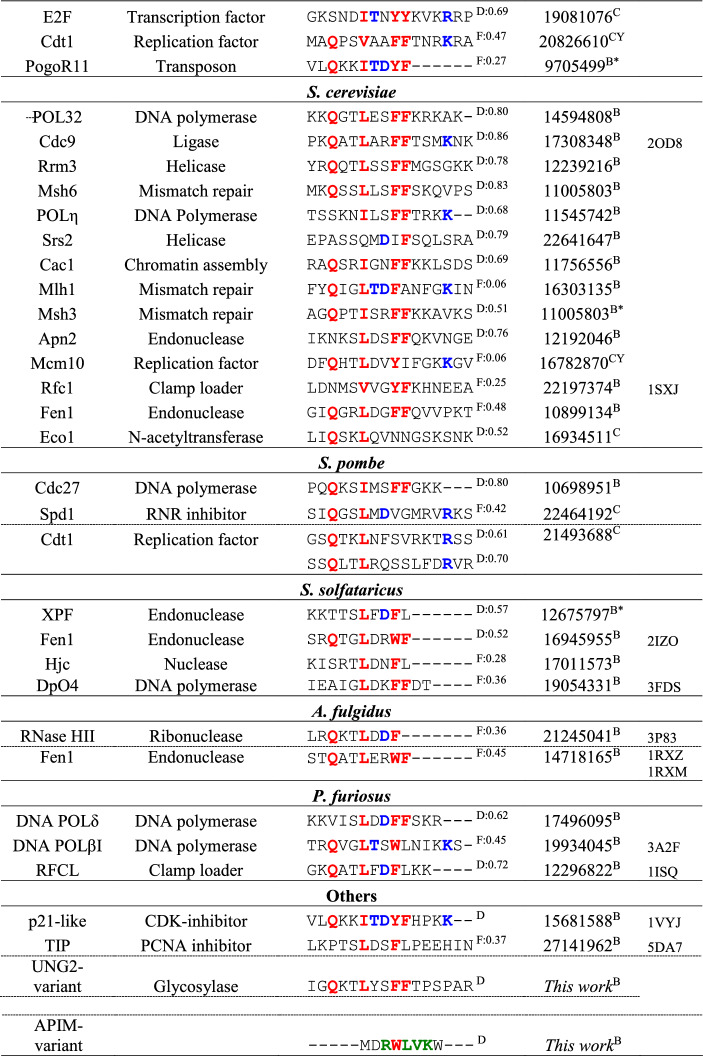
Curated list of PCNA interaction partners

Positions with residues specific to the PIP box motif are marked in red, PIP degron specific in blue, and residues specific to an APIM in green Alignment gaps are marked by -. D/F indicate whether the sequence in predicted to be in a disordered (D) or folded (F) regions, followed by the average disorder propensity, where 1 indicate fully disordered. The method used to determine the interaction is indicated as C (cellular experiments, CY: Yeast-2-hybrid) or B (biophysics in vitro experiments, B* pull-down assays) follows the PMID number

### PCNA-interacting motifs reside in intrinsically disordered regions

**Fig. 3:**
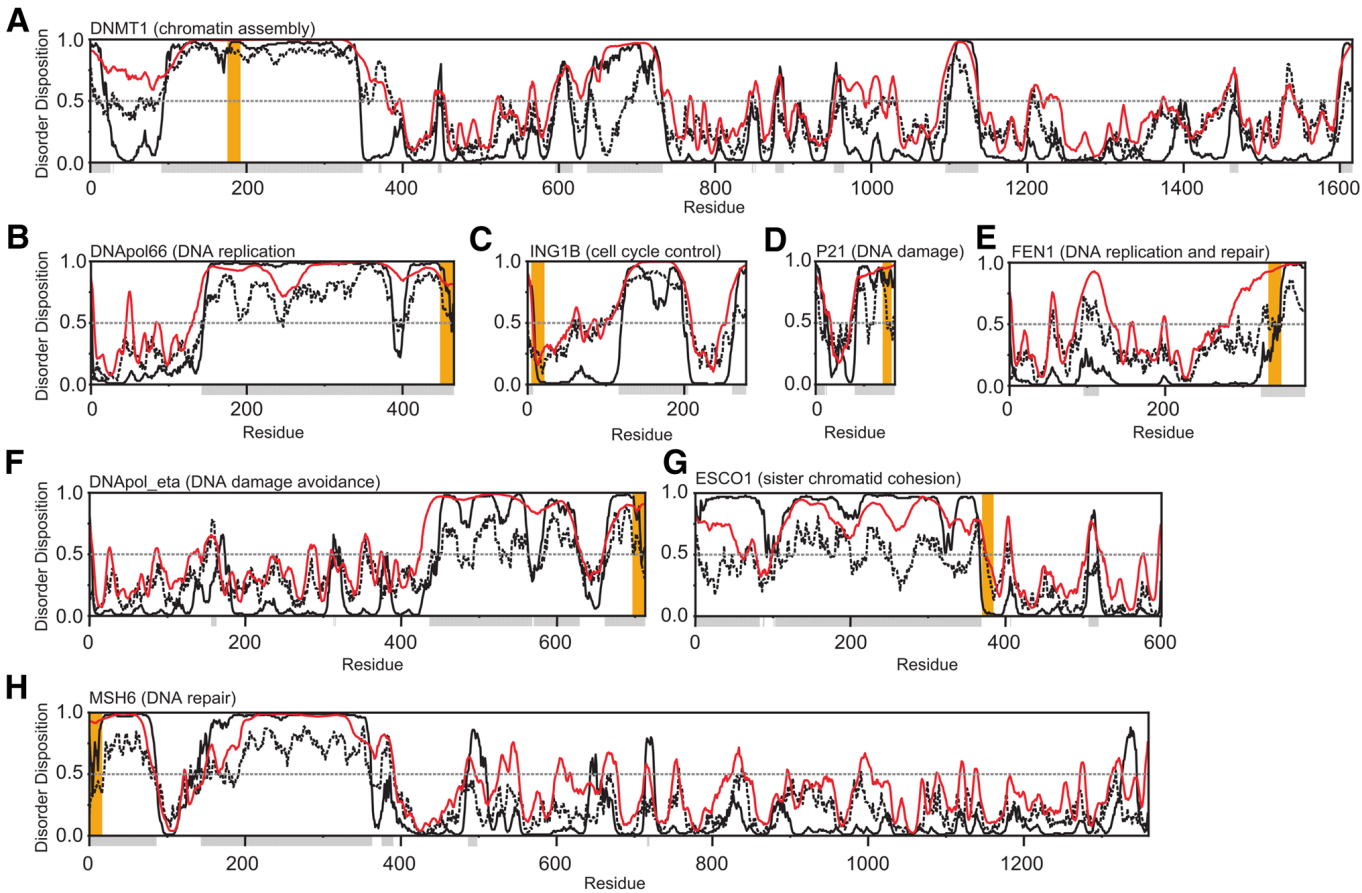
PCNA motifs reside predominantly in intrinsically disordered regions. **a** DNA (cytosine-5)-methyltransferase 1 (human)—chromatin assembly. **b** DNA polymerase δ (human)—DNA replication. **c** p33 (inhibitor of growth 1b) (human)—cell cycle control. **d** p21 (cyclin-dependent kinase inhibitor 1) (human)—DNA damage. **e** Flap endonuclease 1 (FEN1) (human)—DNA replication and repair. **f** DNA polymerase η (human)—DNA damage avoidance. **g***N*-acetyltransferase ESCO2 (human)—sister chromatid cohesion. **h** DNA mismatch repair protein MSH6 (human)—DNA repair. The disorder propensity from 0 to 1 is plotted as a function of residue number and was predicted using Disopred3 (http://bioinf.cs.ucl.ac.uk/psipred) (black), IUpred2 (https://iupred2a.elte.hu) (striped black), and Pondr-fit VSL2 (http://www.pondr.com) (red) and default settings. The disorder for each residue was denoted with gray boxes below the *x*-axis by calculating the average disorder disposition for the three predictors with a threshold equal to or above 0.5 (indicated by gray dotted line). Orange boxes show PCNA binding motif-locations

There are several reports of PIP motifs residing in IDPs [50, 52, 60], or in intrinsically disordered regions (IDRs) [61], but to our knowledge, no systematic analyses of the correlation to disorder have been performed. Using the assembled list of confirmed PCNA binders we wanted to systematically investigate the disorder propensity of the entire sequences of the set of proteins collected in Table 2. Three different bioinformatics tools, Disopred3 [62], IUpred2a [63] and PONDR-VSL2 [64] were used for this purpose and the results of selected examples and the whole set are shown in Fig. 3 and Fig. S2, respectively, and the sequences in Table 2 were annotated according to their disorder propensity. The majority of the wildtype motifs were consistently predicted to be located in IDRs, having an average disorder propensity ≥ 0.5 in all three predictors (64%, 52 motifs). For 13 motifs (16%) at least one prediction tool suggested the motif to be in an IDR, while there was no consistency between the different predictors. In eukaryotic proteins, the PIP-motif can be located at various positions in the sequence, the very N-terminus (MSH6, Fig. 3h), the very C-Terminus (POLD3, Fig. 3b), at the N- or C-terminal edges of folded domains (ING1B, Fig. 3c and FEN1, Fig. 3e) as well as in the middle of long disordered regions (DNMT1, Fig. 3a). In contrast, PIP-motifs in proteins from archaeal organisms *S. solfataricus, P. furiosus* and *A. fulgidus* are all located at the very C-terminus of the sequence indicating in these cases a more restrictive mode of interaction (Table 2, Fig. S2). Some motifs were consistently predicted to reside in ordered regions (20%, 16 motifs) (Fig. S2). Of the 16 proteins where the PIP-motif was predicted to be in ordered regions, structural data of a folded protein bound to PCNA was only available in one case (rfc1 from *S. cerevisiae*, PDB-code: 1SXJ, Fig. S3A). Here the motif is located in a loop region and accessible for PCNA binding in the canonical interaction mode (Fig. 1d). In six cases, a structure of the unbound protein was available and the PIP-motifs were located either within a β-strand (human CHK1, PDB-code: 5WI2; human XPA, PDB-code: 1D4U) or within an α-helix (human DNA pol μ, PDB-code: 4LZD; human DNA pol β, PDB-code: 1BPX; human 14-3-3 ζ δ, PDB-code: 1QJA; *S. cerevisiae* mlh1, PDB-code: 4E4W) and there is no common family of folds apparent. In all these structures (Fig. S3B-G), the hydrophobic residues of the motif are at least partly buried in folded domains and not directly accessible for PCNA binding, and the interpretation of mutational studies are, therefore, convoluted by potential misfolding. To adopt the prevalent binding mode (Fig. 1d), a major structural rearrangement would need to occur, but unfortunately, no structural information of the PCNA-bound states is to date available in these cases.

Collectively, this thorough analysis confirms that flexibility and dynamics provided by structural disorder dominates the majority of PCNA interactors and that the PCNA-interacting motifs exist in such context. Broadly, the disorder is apparent across the different functionalities underscoring that the intrinsic disorder-dependent mode of interaction persists across functions carried out by PCNA.

### The sequence variation of PCNA motifs is large

The binding partners collected in Table 2 are categorized by species and ordered within each species by sequence distance to the motif of p21. Many motifs are seen to resemble the canonical PIP motif at 3 or 4 central positions. Generally, however, and in line with bioinformatics on the APIM [28] there is a striking degree of sequence variation across the set. Although these peptides are all expected to dock into the exact same pockets of PCNA, they deviate considerably both from each other, and from the canonical PIP motif. These variations include a lack of the initial Q in 34% of the sequences, a lack of a hydrophobic residue at position 4 (12%), and a lack of one (43%) or both (10%) of the aromatics at positions 7 and 8. The most promiscuous position appears to be position 1, allowing both negative and positive charged residues as well as Gly and Pro. The most distant sequence that still binds to PCNA is the DNA ligase protein of *S. solfataricus*, containing Glu and Ala in position 1 and 4 and lacking both aromatics at positions 7 and 8. Many of these motifs do not formally adhere to the current PIP-motif sequence specification, which raises the question whether the current definition of the PIP box is too narrow, potentially biased by a historical focus on particular binding partners and the canonical motif.

### Broad structural compatibility of the binding pocket

**Fig. 4:**
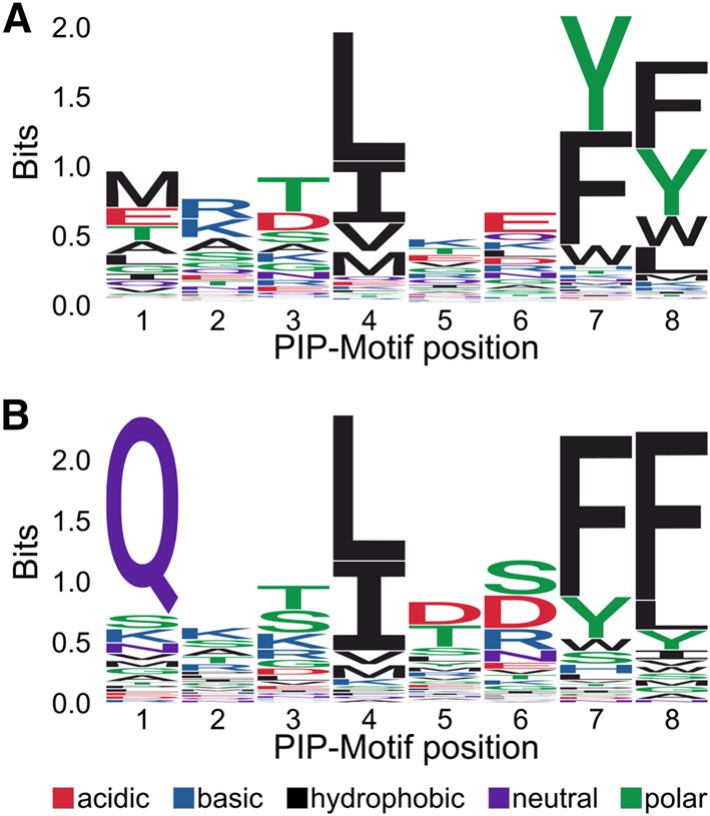
Structure analyses of the PCNA binding pockets across species. **a** Sequence logo of the amino acid distribution predicted by the structural model (letter-height denotes information content). **b** Sequence logo of the amino acid distribution calculated from motifs in Table 2 (letter-height denotes information content)

To investigate whether the current view of the PIP motif is overly restrictive, we revisited the definition of the PIP box from an orthogonal, purely structural perspective, by using a variation of a recent model for predicting the identity of amino acids given the atomic environment surrounding them [65] (See “Methods”). The model treats the atomic environment as a 3D image (separating atom types into different image channels) and uses a convolutional neural network to predict the distribution over the 20 amino acids that is most compatible with such an environment. By conditioning on the specific environment of the PCNA binding pocket in complex with its binding partners, the model allows us to quantify which amino acids are structurally preferred at each position in the motif. Since we were interested in the generic preferences shared across binding partners, we averaged over the available structures of partners in complex with PCNA, Table 2, and the binding sites in each peptide-bound monomer. Although the model omits certain structural details, such as the placement of the side chains of the predicted amino acids, and thus provides only an approximation of the sequence preferences, it agrees remarkably well with the examples listed in Table 2. According to the prediction model, the PIP motif is primarily defined by a hydrophobic residue at position 4, and a Tyr or Phe at positions 7 and 8, panel A of Fig. 4. This computational technique, which is unaffected by any potential selection biases towards particular PCNA binding partners or motif pattern, thus confirms the three anchoring points as the dominant features of the motif, and can thus meaningfully be considered as constituting the canonical motif. The prediction model finds no particular preference for Gln at the first position, which is strongly preferred among known motifs (compare Fig. 4a, b). This could be due to an insensitivity of the model to the specific side chain hydrogen bond interaction that provides stability to the Gln, although purely hydrophobic side chains are also found at this position. While it is feasible that Gln at position 1 provides some degree of energetic stabilization, one could also speculate that its over-representation in recognized interacting partners might originate from a selection bias originating from the current definition of the canonical PIP-box, which lists Q as essential at this position. In an attempt to settle this issue, we made a p21 peptide variant in which we mutated Q at the first position to alanine. By ITC, we measured a 50-fold drop in the affinity for PCNA down to 1.3 μM, thus confirming the important contribution of the Q at the first position (Fig. S4A).

To establish whether the generated logo was stable with respect to the different types of experimental verifications in Table 2, we generated a second logo plot based only on the in vitro biophysics binding studies in Table 2 (marked B in Table 2) as well as including only partner with an annotated disorder > 0.5. No significant differences between these three logos were observed (Fig. S5).

The large differences in binding affinity observed in Table 1 suggest that the residues surrounding the three anchoring sites should be able to dramatically modulate binding affinity. To support this notion, we grafted the flanking regions of the strongest binding peptide, p21 to those of a much weaker binding peptide, Fen1, and measured the affinity by ITC. Indeed, this changed the affinity for PCNA 60-fold to 730 nM (Fig. S4B). The broad sequence variability observed in both Table 2 and Fig. 4a indicates that this type of modulation is not encoded in position specific signals. Therefore, as a potential explanation, we proceeded to investigate the role of charge complementarity between PCNA and its binding partners.

### Net charge of the SLiM-flanking regions correlates to PCNA affinity

**Fig. 5:**
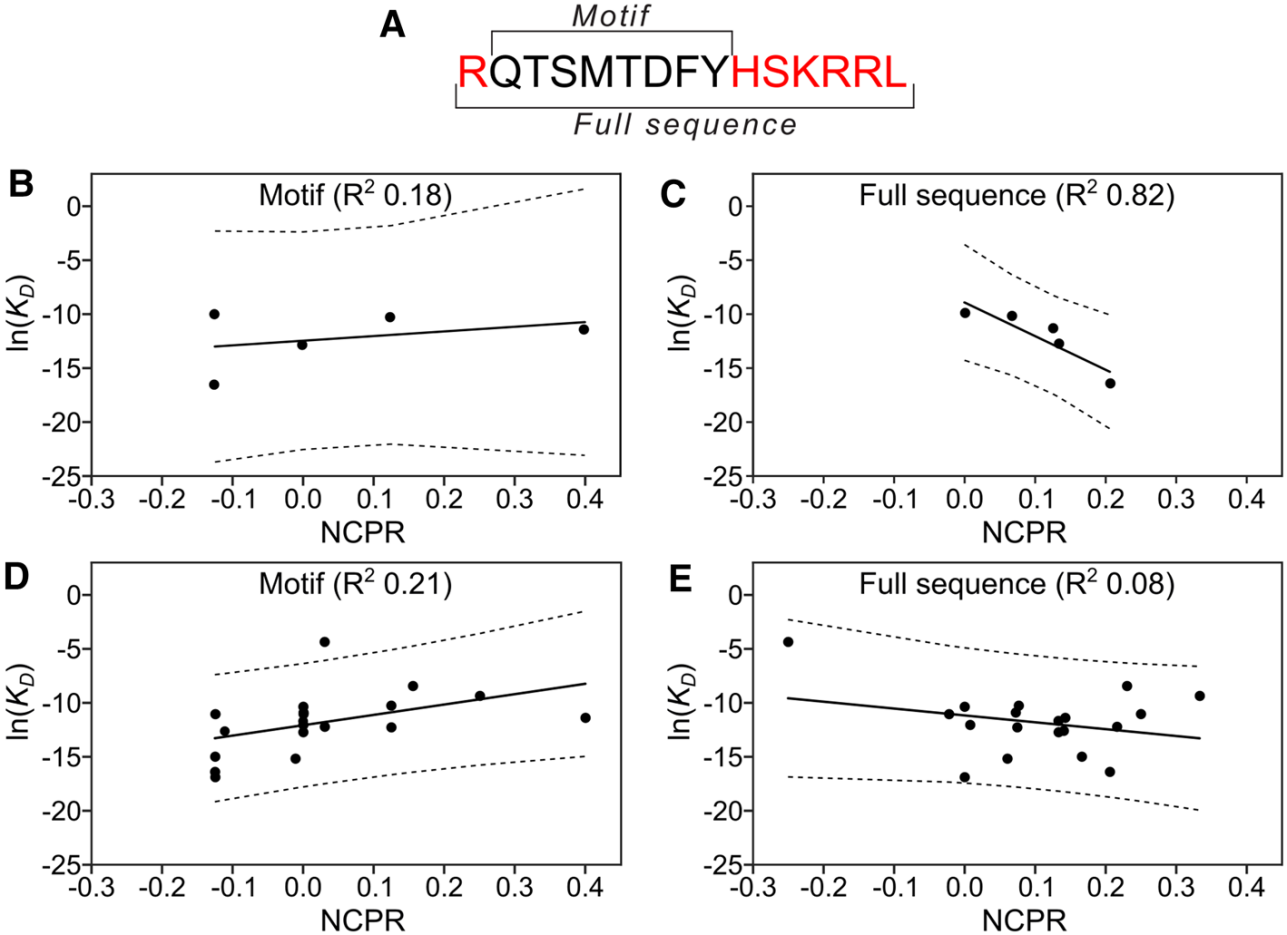
Binding affinities correlate with net charge per residue when features of the flanking regions are included. **a** Sequence of p21 with PIP motif and full sequence indicated. **b** Correlation between PIP motif calculated net charge per residue (NCPR) for Table 1 sequences and their experimental binding affinities. **c** Correlation between full sequence calculated NCPR for Table 1 and their experimental binding affinities. **d** Curated data set (Supplemental Table S2, Figure SX) PIP motif NCPR correlation with experimental binding affinities. In cases where multiple affinities were available for the same protein, an average was used. **e** Curated data set (Supplemental Table S2, Figure SX) full sequence NCPR correlation with experimental binding affinities. In cases where multiple affinities were available for the same protein, an average was used

Electrostatic effects are one of the major drivers of short-range non-covalent protein–protein interactions [66]. The PCNA surface surrounding the binding pockets is highly negatively charged [20] and it was shown that exchanging the flanking regions of p66 (uncharged) to p21 (positively charged) led to a tenfold increase in binding affinity [31], just as grafting the p21 flanks to Fen1 as done in this work increased the affinity by 60-fold. To investigate this effect further, we calculated the sequence-based net charge per residue (NCPR) and correlated it with experimentally observed binding affinities for both the PIP-box itself, and the PIP-box with flanking regions included. For this purpose, we obtained two different lists of binding affinity measurements, one consisting of the direct affinity measurements conducted in the present work (Fig. 2 and Table 1), which are all measured under the same experimental conditions and the other by additionally including available affinity measurements in the literature (Table S2). To allow for a meaningful comparison across binding partners we chose a fixed length of seven residues of the flanking region (Fig. 5a). In some cases, the lengths of the studied proteins were shorter than this flanking region, resulting in shorter effective flanking regions. To make sure this did not affect our analysis we redid our experiments with these cases excluded (Fig. S6A and B). The NCPRs were calculated at the pH of the respective experimental affinity measurement for the different sequences in Supplemental Table S2.

The regression analysis using our experimentally determined affinities showed a significant correlation between NCPR and binding affinity when the flanking regions are included, while the charges of the PIP-box region alone hardly correlate with affinity (displaying a slight positive correlation) (Fig. 5b, c). The equivalent plot using affinities from the literature did not display the same pattern (Fig. 5d, e, Fig. S6C and D). We hypothesize that this is due to the many different experimental conditions used in the literature-based affinity measurements, including variations in temperature, pH, and the fact that some of the entries are recorded on full-length proteins that have additional contacts to PCNA/DNA [67]. For example, the entries for Fen1 deviate three orders of magnitude even for two very similar peptides. To probe the effect of the flanking regions even more clearly, we proceed with an experiment where the motif is kept completely fixed while only the flanking regions are modulated.

### Charge modulation of flanking regions affect PCNA affinity in a predictable manner

**Fig. 6:**
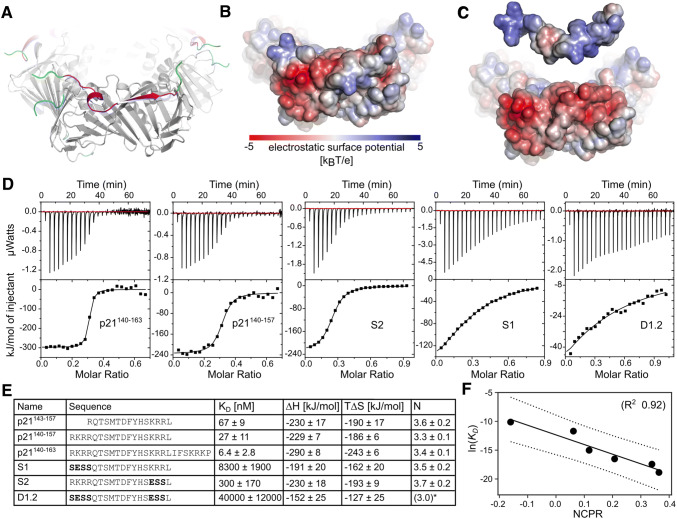
Modulating affinity by modulating flaking region features. **a** Ribbon cartoon representation of the p21-PCNA-complex (PDB-code 1AXC) focusing on the outwards facing surface of the ring and one PCNA monomer. PCNA is colored in gray, p21 in magenta and modeled stretches not visible in the crystal structure (p21^139–142^, p21^161–164^, PCNA^107–108^, PCNA^187–190^ and PCNA^256–261^) are highlighted in green for p21 and cyan for PCNA, respectively. **b**, **c** Electrostatic potential mapped onto the surface of the p21-PCNA complex (**b**) and the separated individual components (**c**). **d** ITC analysis of p21-variants binding to human PCNA. The upper portion shows baseline-corrected raw data from the titration, and the lower portion shows the normalized integrated binding isotherms together with the fitted binding curves fitted to a “one set of sites” model. All experiments were carried out in triplicates and representative ITC measurements for injection of PCNA into each peptide are shown. *N was fixed to 3 to achieve convergence of the fit. **e** Table of names, sequences, and ITC-obtained thermodynamic parameters of p21-variants. Residues deviating from the sequence of wildtype p21 are bold. **f** Correlation of ln(*K*_D_) vs NCPR for the different p21 variants in **e**

The highly negatively charged PCNA surface patches surrounding the binding pocket are complementary to positive stretches (^140^RKRR^143^, ^154^KRR^156^, and ^161^KRK^163^) in the flanking regions of the p21 peptide (Fig. 6a–c). To test if these electrostatic interactions are the reason for the much higher affinity of p21 towards PCNA compared to other canonical PIP-motifs, we concluded our study by designing a number of p21-based peptides, explicitly modulating the overall charge of the flanking regions, and measuring their binding affinity to PCNA. First, the length of the flanking regions was increased at both ends to include either one additional positive stretch at the N-terminus (p21^140–157^) or all three positive stretches (p21^140–163^). Second, we systematically reduced the net charge of the p21^140–157^ peptide by mutating the arginine and lysine residues of the positive stretch at the N-terminus (^140^RKRR^143^) or the C-terminus (^154^KRR^156^) or both, to hydrophilic (Ser) or negatively charged (Glu) residues, respectively, yielding ^140^SESS^143^ and ^154^ESS^156^. As the capturing antibody showed unspecific binding, some of these peptides were not suitable for analysis by SPR and affinities of all p21 variants were thus determined using ITC. We found that including the residues ^140^RKR^142^ in the p21 peptide and thereby increasing the net positive charge, lowered the *K*_D_ from 67 ± 9 nM for the initial p21 peptide to *K*_D_ = 27 ± 11 nM for the extended peptide. When both basic regions ^140^RKR^142^ and ^161^KRK^163^ were present, the affinity of the entire peptide increased further to a *K*_D_ = 6.4 ± 2.8 nM (Fig. 6d, e). In line with our hypothesis, any variants with a lower NCPR had a strongly decreased affinity for PCNA (Fig. 6d, e) and we see a strong negative correlation of ln(*K*_D_) vs NCPR (Fig. 6f). Thus, despite all p21 peptide variants having identical PIP motifs, the affinities span four orders of magnitude, and vary systematically with the charge propensities of the flanking regions, highlighting how affinity modulation can be obtained through changes of the charges in the flanking regions.

## Discussion

PCNA is an important cellular hub with an extensive interactome, involved in maintaining fidelity in DNA-related processes as well as processes in the cytosol. Interaction with PCNA occurs through three identical sites on the surface of the PCNA homotrimer and is believed to rely critically on the presence of specific SLiMs in the binding partners. The best described of these is the PIP-box, but other SLiMs have recently been identified to bind to the same binding site. The PIP-box motif is typically defined as having a Gln at the first position, an aliphatic hydrophobic residue at the fourth position, and two aromatics at positions seven and eight. However, deviations from these requirements have been observed (Table 2) and the determining factors for binding to PCNA have remained controversial [68]. In this study, we aimed to address this issue systematically by various complementary approaches including direct affinity measurements and correlation of *K*_D_ to biophysical properties, which overall pointed to charges in the motif-flanking region having an essential contribution to binding affinity. Finally, this hypothesis was experimentally validated by systematically reducing charges in the flanking regions of the motif of p21, allowing us to modulate the affinity of PCNA binding by four orders of magnitude.

### Charges in the flanking regions modulate PCNA affinity

Our curated set of PCNA binding partners from the literature demonstrates clearly that the sequence of the binding motif is highly diverse and mostly located in disordered regions, suggesting that the PCNA binding site itself is fairly tolerant, and not overly specific. We investigated if deviations from the canonical PIP-motifs are associated with a decrease in binding affinity, but found no evidence to support this, since strongly degenerate sequences like the APIM peptide bind the same pocket with affinities comparable to many canonical PIP-motifs. To address whether other positions within the PIP-motifs could play a critical role, we conducted a computational analysis using the available three-dimensional structures, the result of which primarily restated the known sequence demands at sites 4, 7 and 8, while the importance of Gln at the initial position was confirmed with ITC. Finally, using NMR spectroscopy, we investigated an earlier claim that preformation of secondary structure within the motif would have a significant impact on binding affinity [49, 51, 52], but find no support for this. Collectively, these results point to a substantial role of the flanking regions surrounding the SLiM. Calculations of charge complementarity between PCNA and its binding partners support this idea. To directly test the effect, we constructed a set of binding partners with the canonical PIP-motif of p21 combined with charge-altering mutations in the flanking regions and observe a remarkably clear correlation between positive charges in the flanking regions and a strong affinity. Importantly, the correlation between binding affinity and NCPR increased when going from binding data that included different motifs measured at several different conditions (Fig. 5d), over different motifs measured under the same conditions (Fig. 5b), to the same motif with varying flanking regions, also at same conditions (Fig. 6f). The latter two figures are overlaid in Fig S7A, where it is apparent that different motifs will likely follow diffent slopes, depending on an interplay of variations in core motif positions and flanking regions.

Overall, and in line with earlier observation our results suggest the interaction between PCNA and its binding partners extends considerably outside what is typically characterized as the binding motif. The extent by which these regions can modulate affinity is shown here under controlled experimental conditions to span over four orders of magnitude. It is not surprising that the binding affinity is not encoded exclusively in three or four sites, since such patterns would arise frequently by chance. One might have expected that a canonical motif would be a necessary (but insufficient) criterion for binding, but the sequence diversity observed in our curated set of binding partners suggests otherwise. While the motif itself certainly has strong sequence preferences at specific positions, there is not any single position that is perfectly conserved, and we even see extreme examples of binding partners with only one canonical residue [e.g., Srs2 (ASSQMDI**F**)] or with shorter sequence span [e.g., CAF1 (**Q**AR**L**–PF)]. Our findings have distinct implications for the identification of new PCNA binding partners; searching for canonical PIP-motifs will leave many potential candidates uncovered, and some candidates that are found this way could prove to be false positives due to incompatible flanking regions. It also provides a frame for interpretation of disease-related mutation, either in PCNA or its binding partners; a cancer variant of human ABH2, mutated near its APIM motif at position + 5 (Q − > K), has enhanced affinity for PCNA [69].

### Are SLiM-flanking regions generally important?

It is conceivable that various diffuse features in the flanking regions (such as net charge) play a crucial role for many SLiM-based interactions and should be considered as an equal contributor besides the usual site-specific amino acid preference defining a SLiM. Several earlier studies have also found flanking regions to play a role in modulating binding affinity [31], also for ligands not binding to PCNA. Based on the ELM database, peptide-based interactions were analyzed and the context found to contribute on average with 21% of the binding energy, as well as being a crucial factor in determining specificity [70]. In particular, it appears that modulation of flanking charges is especially well suited for tuning affinity. Proteins carrying proline-rich motifs bind to SH3 domains, and positive charges in the flanking region modulate the affinity [71–73]. Similarly, for pocket proteins binding the SLiM LxCxE, negative charges in the flanking regions act as affinity and specificity modulators [74]. However, the extent by which a motif’s binding affinity can be modulated via a flanking region (here by four orders of magnitude) has, to our knowledge, not been reported previously. Posttranslational modifications such as phosphorylation and acetylation are regulatory means by which NCPR can be further modulated. Indeed, phosphorylation of p21 at the 2-position of the motif has shown a strongly modulated PCNA binding [75]. Likewise, the accessibility of the binding surface on PCNA may by itself contribute to selectivity. Thus, either by posttranslational modification or by screening from binding of other proteins participating in DNA homeostasis, PCNA may indirectly regulate the degree by which the ligand binding flanking regions may contribute to binding. This has to the best of our knowledge, not yet been addressed.

### One common class of PCNA motifs with sub-group idiosyncrasies

Traditionally, PCNA-interacting motifs have been classified as PIP-boxes, PIP-degrons and APIM-motifs. Given the large sequence diversity within the PIP-box motifs, one could pose the question whether these three motifs are fundamentally different, or whether they are all samples from a larger population? The PIP-degron is known to be very similar to the PIP-box but requires a *K*/*R* at the + 4 position downstream from the motif and often carries a TD at position 5 and 6 [27]. The APIM has larger sequence divergence, but still harbors a hydrophobic residue at position four and an aromatic/hydrophobic at sites 7 and 8 and is thus not more at odds with the canonical PIP motif than other sequences in our curated set. Furthermore, recent crystal structures show that APIM binds in a very similar mode to the PIP-box motif [47, 48]. Both the APIM and PIP degron have requirements for specific amino acids at sites slightly downstream from the standard PIP-box motif, which seems to be related to their specific functions [27, 28]. Interestingly, we note that these additional requirements increase the NCPR and hence could compensate for motif divergence. Thus, in chimeras made from degron, boxes and APIMs, focusing solely on exchanging the motifs, flanking regions are not transferred, which make interpretations of the resulting functional effects complicated [76]. It is also likely that many of the PIP-motif examples in Table 2 encode additional site-specific residue preferences related to their other function(s), thereby modulating the motif’s canonicity. It might, therefore, be beneficial to consider PIP-box motifs, PIP-degron motifs, and APIM motifs as members of the same broad class of PIP motifs (defined using position 4, 7, and 8 in the current PIP box definition), while accepting that every single motif carries its own idiosyncrasies determined by all its functions. Such idiosyncrasies might be evolutionarily conserved for binding partners with similar functionality, which implies that PIP-motifs possibly can be divided into function-related subclasses, of which the APIM and the PIP-degron constitute relevant examples. According to this view, the PIP-degron can be considered as a sub class that can also accommodate the interaction with the CRL4-Cdt2 E3 ubiquitin ligase [77].

### A broader view on SLiMs?

Current estimates suggest there may be in the order of 1,000,000 different SLiMs in the human proteome [4]. However, despite their abundance and importance, far fewer have been properly described. Our work suggests that we may be able to take a broader view on SLiMs, which includes the flanking context, an expansion that may help us to define new SLiMs more rigorously, which is currently a very tedious and experimentally challenging task. In the case of PCNA, the focus on positive charges may allow us to find more—and more degenerate—motifs, which are currently not being predicted. From our work on the modulation of the charges of the p21 peptide, it appears that we may use the NCPR as an approximate ruler for predicted affinity. Thus, motif-containing proteins with negatively charged flanking regions likely do not bind to PCNA. It is also possible that a trade-off exists between modulation of the motif sequence as a result of intertwined binding sites and modulation of the flanking regions, such that less hydrophobic motifs are compensated by an increased number of positive charges in the flanking regions. To this end, we calculated the corresponding NCPR for different lengths of the flanking regions (Fig. S7B). This revealed a strong preference for positive charges up to a flanking length of ± 5 residues. These flanking regions are lacking in most crystal structures where either only trimmed peptides were used or because of flexibility in the bound state. For p21, the N-terminal flank (^140^RKRR^143^) is disordered and not visible in crystal structures, but NMR showed that it still makes contacts to C-terminal residues of PCNA [46] as apparent in strong NMR chemical shift perturbations. These PCNA residues are mainly negatively charged, disordered and invisible both in the free form and in the complex structure. The positively charged residues (^154^KRR^156^) of the C-terminal flank of p21 are still visible in the complex structure (pdb-code: 1AXC) and show multiple stable contacts, where the head-group of R155 is positioned between the negative charged residues D122 and E124 in the IDCL of PCNA and R156 is involved in a salt bridge to D29. The last positive stretch (^161^KRK^163^) was not part of the construct. Whether or not an optimal length exists for contributing flanking regions and if this is evolutionary conserved remains to be addressed.

## Conclusion

In this work, we challenge the view of the PIP-box as the dominant determinant for binding. Through a systematic review of known experimentally validated binding partners for PCNA, we demonstrate that there is a substantial divergence from the canonical motif among known binding partners. As a complementary source of binding affinity, we suggest that the flanking regions surrounding the PIP-motifs play an essential and modulatory role. We provide substance to this claim by systematically manipulating charges in flanking regions around the motif of p21 and demonstrate that this allows us to modulate binding affinity over four orders of magnitude. This provides an explanation for the large diversity within the PIP motifs of known PCNA binders and suggests directions for the search for new interaction partners for PCNA. Furthermore, we anticipate that the ability to engineer the affinity to this extent opens new possibilities in drug development.

## Materials and methods

### Protein expression and purification

Expression and purification of Spd1 was based on previously published protocols [60]. N-terminally His_6_-tagged human PCNA [40] in a pQE32 vector was expressed in *Escherichia coli* (*E. coli*) BL21(DE3) cells in LB-Medium at 37 °C. The full sequence of the N-terminal tag is MGSSHHHHHHSSGLEVLFQGPH. Protein expression was induced at an OD_600_ of 0.6–0.8 with 0.5 mM IPTG and cells were grown for 4–6 h at 37 °C. Harvested cells were resuspended in lysis buffer [50 mM Tris, 150 mM NaCl, 20 mM imidazole, pH 8.0, complete EDTA free protease inhibitor cocktail tablet (Sigma-Aldrich)], and lysed by passage through a French press at a pressure of 1100–1400 Psi. The cell debris was removed by centrifugation at 30,000*g* for 50 min at 4 °C, and the supernatant incubated with 3–4 mL Ni^2+^-NTA resin (GE Healthcare Life Sciences) for 1 h at room temperature on a tilting table prior to being applied to a gravity column. The column was washed with 20 column volumes of washing buffer (50 mM Tris, 1 M NaCl, 20 mM imidazole, pH 8.0), followed by 10 column volumes of lysis buffer, and eluted using 4 column volumes of elution buffer (50 mM Tris, 250 mM imidazole, pH 8.0). The eluate was concentrated using a 10000 MWCO filter (millipore) prior to being applied to a size-exclusion chromatography column (HiPrep Sephacryl S-200 16/60 or HiLoad 16/600 Superdex 200) pre-equilibrated with purification buffer (10 mM NaH2PO4, 100 mM NaCl, 2 mM DTT, pH 7.0). The sample was eluted in purification buffer using an ÄKTA prime or ÄKTA pure 25 chromatography system. Purity was checked by sodium dodecyl sulfate–polyacrylamide gel electrophoresis (SDS-PAGE) and homogeneity checked by dynamic light scattering. Relevant fractions were pooled and stored at 5 °C if used within 14 days or otherwise flash frozen and stored at − 20 °C until further use.

### Peptides

All peptides were at > 95% purity and, except the UNG2 variant, were N-terminally acetylated and C-terminally amidated. A total of 12 different peptides were used: seven variants of human p21, hereof three wildtype variants (p21^143–157^, p21^140–157^, p21^140–164^ Ac-RKRRQTSMTDFYHSKRRLIFSKRKP-NH_2_) and four mutant variants of p21^140–157^ (S1 (Ac-SESSQTSMTDFYHSKRRL-NH_2_), S2 (Ac-RKRRQTSMTDFYHSESSL-NH_2_), D1.2 (Ac-SESSQTSMTDFYHSESSL-NH_2_) and p21^140–157,Q144A^ (Ac-RKRRATSMTDFYHSKRRL-NH_2_); three peptides derived from human wildtype proteins, MSH6 (Ac-RQSTLYSFFPKSPAL-NH_2_), FEN1 (Ac-TQGRLDDFFKVTGSL-NH_2_), and an UNG2 variant (MIGQKTLYSFFTPSP), as well as a synthetic peptide harboring an APIM (Ac-MDRWLVKW-NH_2_) and a chimeric peptide comprising of the Fen1 motif and the flanking regions of p21 (Ac-RKRRQGRLDDFFHSKRRL-NH_2_). For SPR or ITC the peptides were dissolved in water pH adjusted and lyophilized prior to use. Concentrations of peptides were determined by absorbance at 280 nm using calculated absorption coefficients (https://web.expasy.org/protparam/), or, in the case of FEN1, by 1D-^1^H-NMR spectroscopy using DSS as a standard of known concentration.

### Selecting and curating reported PCNA binding partner using a PCNA motif

PCNA binding partners listed in this paper were all reported to bind through a PIP-box, a PIP-degron, or an APIM and were from *H. sapiens, D. melanogaster, S. cerevisiae, S. pombe, S. solfataricus,* or *P. furiosus*. PCNA binding partners were included only if binding through the motif was confirmed. The motif was considered to be confirmed either when (1) mutations of residues of the motif, or deletion of the region of the protein containing the PIP-motif (max 50 residues) led to decreased affinity of the binding, or (2) if a crystal structure of the PCNA-protein complex had been solved or (3) the binding of peptides of a maximum length of 50 residues that included the motif. Methods that were considered to confirm binding (or a decreased binding affinity) were biophysical in vitro experiments, pull-down assays with purified proteins/peptides, and dot plots using purified peptide (marked jointly with B in Table 2), pull-down assays with at least one interaction partner being in the lysate (marked with B* in Table 2), cellular assays where degradation is inhibited (marked with C in Table 2), and yeast-two-hybrid assay (marked with CY in Table 2). Of the 83 PCNA motifs in Table 2, 34 were originally mentioned in Moldovan et al. [25]: here 31 were listed as confirmed PIP boxes by, 2 were listed as putative PIP box, and 1 was listed as a PCNA-binding protein without a proposed PIP box.

### Disorder predictions

Prediction of structural disorder in the curated set of PCNA-interacting proteins was done using three different predictors, Disopred3 (http://bioinf.cs.ucl.ac.uk/psipred), IUpred2 (https://iupred2a.elte.hu), and Pondr-fit VSL2 (http://www.pondr.com) using default settings. The disorder for each residue was assigned by calculating the average disorder disposition for the three predictors with a threshold equal to or above 0.5

### NMR experiments

#### Assignments and secondary structure propensities

NMR samples of Spd1 were prepared by adding 10% (v/v) D_2_O, 0.02% (w/v) sodium azide and 0.5 mM DSS to 309 μL of a 50 μM protein solution in 10 mM NaH_2_PO_4_ and 100 mM NaCl (pH 7.4), giving a final concentration of 44 μM Spd1. The pH of the sample was checked and regulated to 7.4 using NaOH and HCl if needed. p21^143–157^, FEN1, MSH6, APIM, and UNG2 samples were prepared by dissolving the peptides in 2 mM dithiothreitol, 1.25 mM DSS and 5% (v/v) D_2_O and adjusting pH to 6.3 by addition of NaOH. To remove insoluble material, the samples were centrifuged for 5 min at 20,000×*g*, before being transferred to a 5 mm Shigemi NMR tube or a 3 mm Precision NMR Sample Tube (Wilmad). A set of triple-resonance NMR spectra for the assignment of Spd1 was recorded on a Varian Unity Inova 750 or 800 ^1^H MHz NMR spectrometers equipped with a room temperature probe with Z-field gradient; pulse sequences used were from the Varian Biopack. The spectra of the peptides were recorded on a Varian Unity Inova 750 MHz spectrometer equipped with a Bruker TCI cryogenic probe or a Bruker AvanceIII 600 MHz spectrometer equipped with Bruker TCI cryogenic probe. For Spd1, ^1^H-^15^N-HSQC [78] spectra and triple-resonance spectra, HNCACB [79], CBCACONH [80], HNCO [81], HNCACO [82], were recorded at 4 °C. For p21^143–157^, FEN1, MSH6, UNG2, and APIM ^1^H^−1^H-TOCSY [83], ^1^H^−1^H-ROESY [84], ^1^H^−1^H-DQF-COSY [85] and ^1^H^−13^C-HSQC [86] spectra were recorded at 25 °C. All spectra were referenced to DSS in the ^1^H direct dimension and the ^15^N and ^13^C dimensions indirectly using the gyromagnetic ratios. All spectra were zero-filled, apodized using a cosine bell window function in all dimensions, Fourier transformed, and phase corrected manually using either TopSpin^®^3.5 pl 5, nmrDraw, a component of NmrPipe [87] or qMDD [88] if spectra were recorded using non-linear-sampling (NLS). All spectra were analysed and assigned manually in the CCPNmr Analysis software [89]. Secondary chemical shifts were calculated by subtracting the random coil chemical shifts [56, 57] from the experimentally obtained chemical shifts. The NMR chemical shifts for p15^PAF^ were obtained from the BMRB entry 19332 [50].

#### Surface plasmon resonance (SPR) experiments

A stock of > 100 µM PCNA in purification buffer was diluted to the working concentration of 200 nM in 10 mM NaH_2_PO_4_, 500 mM NaCl, pH 7.0, 1 mM β-mercaptoethanol. The peptides were dissolved in SPR running buffer (i.e., 10 mM HEPES, 150 mM NaCl, 0.05% (v/v) P20, pH 7.4).

Binding analyses were recorded on a Biacore T200™ instrument (GE Healthcare Life Sciences). A single cycle protocol setup was used for the injection of ligands in flow cells with PCNA captured by an anti-His antibody covalently coupled to a CM5 chip (GE Healthcare Life Sciences, Cat# BR100530). The carboxymethylated dextran surface was preactivated by injecting 35 µL of a freshly prepared solution of 0.48 M *N*-ethyl-*N*-(3-(diethylamino)propyl)-carbodiimide (EDC) and 0.1 M *N*-hydroxysuccinimide (NHS). The anti-histidine antibody (GE Healthcare Life Sciences, Cat#: 28995056) was immobilized to the activated dextran surfaces of both flow cells by injecting 50 µL of 6–50 µg/mL anti-his antibody in 7.5 mM NaCl, 9.5 mM sodium acetate, pH 4.5, yielding coupling levels of 3750–14000 RU. Remaining activated NHS-esters were blocked by injecting 35 µL 1 M ethanolamine, pH 8.5. All procedures were conducted at a flow rate of 5 µL min^−1^. The binding surface was prepared by loading 30 µL 200 nM PCNA at a flow rate of 10 µL min^−1^, which typically led to capture levels of 500–1800 RU PCNA corresponding to 5–19 fmols mm^−2^ on the anti-His antibody. The control surface was not exposed to PCNA. Each single-cycle protocol consisted of five sequential injections of twofold serial dilutions of the analyte over both flow cells with a contact time of 90 s, and a dissociation time between injections of 225 s, at a flow rate of 50 µL min^−1^. The injection cycles alternated between injections cycles of buffer and peptide with no intervening regeneration. For p21 the final concentrations of the injection cycles were 50 nM and 200 nM, for MSH6, FEN1, UNG2 and APIM, the concentrations of the final injection ranged from 12.5 to 120 µM. For MSH6, the injection cycle with a final concentration of 12.5 µM was repeated as a final injection cycle. For the UNG2, APIM, and FEN1 measurements, a final injection cycle of p21 with a final concentration of 50 nM was run as a control of consistency. Complete regeneration of the flow cells between each single-cycle run was accomplished by injecting 10 mM glycine–HCl, pH 1.5 for 60 s with a flow rate of 30 µL min^−1^. All measurements were carried out at 25 °C.

#### Isothermal titration calorimetry (ITC)

PCNA was buffer exchanged and concentrated using a 10000 MWCO filter (millipore) against the ITC buffer (10 mM HEPES, 150 mM NaCl, 1 mM EDTA, 1 mM TCEP, pH 7.4) for all experiments. The lyophilized peptides were dissolved in the ITC buffer at concentrations between 0.2 and 1 mM determined using A_280_. For all experiments, PCNA was injected into peptide. For p21^143–157^ the peptide concentration was 10 µM, and the PCNA trimer concentration 40 µM. For p21^140–157^ and p21^140–163^, the peptide concentration was 10 µM, and the PCNA trimer concentration 36 µM. For experiments with S2, S1, and D1.2, the peptide concentrations were 20 µM, 60 µM, and 50 µM, respectively, and the PCNA trimer concentration 100 µM, 200 µM, and 269 µM, respectively. All buffer, protein, and peptide solutions were properly degassed prior to loading. ITC was performed with the microCal instrument ITC200 (Malvern). Data were recorded at 25 °C with a stirring speed of 307 rpm and a reference power of 10 µCal s^−1^. The heat of dilution was determined from the final injections after saturation of the complex and was subtracted from the data for all peptides except S1 and D1.2 where saturation was not reached. Data from the ITC experiments were analyzed using the Origin 7 software package (MicroCal™). The data were fitted to a single binding site model.

#### Structure modeling

A model of the PCNA-p21 complex was built to include the lacking dynamic regions (Fig. 6a–c) and was generated based on the crystal-structure (PDB-code: 1AXC). The dynamic regions not visible in the crystal structure (p21^139–142^, p21^161–164^, PCNA^107–108^, PCNA^187–190^ and PCNA^256–261^) were modeled using the webserver ModLoop [90] (software available at: https://github.com/salilab/modloop). All structures are visualized using PyMOL™ Molecular graphics system, version 1.8.0.0 (^©^Schrodinger, LLC). The hydrophobicity of the PCNA surface in Fig. 1c was colored using the residue-specific hydrophobicity scale according to Eisenberg et al. [91] (Source: http://us.expasy.org/tools/pscale/Hphob.Eisenberg.html). The electrostatic surface of the PCNA-p21 complex in Fig. 6b, c was generated using the APBS plugin version 1.4 for PyMOL (APBS-software available from: https://github.com/Electrostatics/apbs-pdb2pqr) [92].

#### Convolutional neural network

The original implementation for the convolutional neural network (CNN) for structure-based amino acid prediction is available at https://github.com/deepfold/. For the present work, we employed a faster implementation, which differs primarily in its input representation. In the original publication [65], the network used a two-channel representation encoding partial charge and mass for each atom. In this new iteration, the network makes use of a five channel one-hot encoding representation, one per atom type expected in the dataset. The spherical-polar coordinate projection was used, with the same layer configuration as the original publication, apart from the number of channels in the first layer.

The CNN was trained using the same dataset as the previous publication. It contains 2336 proteins, filtered to a maximum of 30% sequence homology pairwise. A 90% and 10% division was made for training and testing, respectively. The training set was further divided into training and evaluation sets, with 1891 proteins used in testing and 211 for evaluation, corresponding to 10% of the full training set. Finally, the testing set consisted of 234 proteins.

The PCNA-bound PIP box instances in Table 2 for which PDB structures were available were downloaded and prepared by projecting each residue’s environment onto the spherical-polar coordinate representation. Due to differences between hetero- and homotrimers in the dataset, as well as different structural determination techniques, we include only one of the PIP box monomers for each structure. Each PIP box Cα-_α_position was then used as the basis of the amino acid prediction for that specific residue index, with the overall probability average being calculated over all structures’ predictions for the same position. Finally, from the resulting probability distribution, and using the ggseqlogo plugin for R (https://github.com/omarwagih/ggseqlogo) [93], we plot the structural PIP box prediction as shown in Fig. 4.

### N- and C-terminal NCPR histograms

Net charge per residue was calculated using localCIDER for each studied sequence. These were generated by including the PIP-box sequence and adding different neighboring-residue lengths around the PIP-box. We included between 1 and 9 residues on each side, totaling a maximum of 18 residues added. Only entries in which the protein sequence has residues in all positions were considered in the NCPR averaging.

### Regression analysis

Net charge per residue (NCPR) was calculated using localCIDER (http://pappulab.github.io/localCIDER/) [94], a Python 3 library for disordered protein analysis from sequence. The subsequent regression analysis was performed using the StatsModels library in Python 3 (https://www.statsmodels.org).

### Quantification and statistical analysis

#### Surface plasmon resonance

Fitting of the data by non-linear regression to a bimolecular interaction model, assuming pseudo-first order reaction condition yielded the association (*k*_on_) and dissociation (*k*_off_) rate constants. The actual rate constants were only derived for p21^143–157^ as the association and dissociation rates for the weak binders were too fast to be reliably quantified. In these cases, only *K*_D_s were obtained by fitting responses at equilibrium, *R*_ss_ to the equation $R_{\text{ss}} = \frac{{K_{\text{A}} \cdot c \cdot R_{\rm{max} } }}{{K_{\text{A}} \cdot c \cdot n + 1}}$ where c is the peptide concentration at which the binding is observed, *n* is the stoichiometry of the binding and *R*_max_ is the predicted maximum response for that amount of PCNA. For runs with different capture levels of PCNA for each injection cycle, the responses were normalized to a common *R*_max_ value. We used the evaluation software supplied with the instrument for global fitting (BiacoreT200 Evaluation 3.0).

#### Isothermal titration calorimetry

All experiments were carried out in triplicates. The *K*_D_, Δ*H* and *n* reported in the text and Fig. 6b are the mean of the values extracted from each measurement, with the standard deviations calculated using error propagation of the standard errors of the fits for the individual measurements. TΔS values reported in the text and Fig. 6b are the mean of the values extracted from each of the measurements, and the standard deviations are of the three independent measurements. In the case of the D1.2 peptide, the stoichiometry was kept constant at three peptides per PCNA molecule to achieve convergence in the fitting procedure.

### Electronic supplementary material

Below is the link to the electronic supplementary material.

Supplementary material 1 (DOCX 7507 kb)

## References

[1] O’Shea C, Staby L, Bendsen SK (2017). Structures and short linear motif of disordered transcription factor regions provide clues to the interactome of the cellular hub protein radical-induced cell death 1. J Biol Chem.

[2] Cumberworth A, Lamour G, Babu MM, Gsponer J (2013). Promiscuity as a functional trait: intrinsically disordered regions as central players of interactomes. Biochem J.

[3] Van Roey K, Uyar B, Weatheritt RJ (2014). Short linear motifs: ubiquitous and functionally diverse protein interaction modules directing cell regulation. Chem Rev.

[4] Tompa P, Davey NE, Gibson TJ, Babu MM (2014). A Million peptide motifs for the molecular biologist. Mol Cell.

[5] Chen JW, Romero P, Uversky VN, Dunker AK (2006). Conservation of intrinsic disorder in protein domains and families: II. Functions of conserved disorder research articles. J Proteome Res.

[6] Davey NE, Van Roey K, Weatheritt RJ (2012). Attributes of short linear motifs. Mol Bio Syst.

[7] Davey NE, Cyert MS, Moses AM (2015). Short linear motifs—ex nihilo evolution of protein regulation. Cell Commun Signal.

[8] Gibson TJ, Dinkel H, Van Roey K, Diella F (2015). Experimental detection of short regulatory motifs in eukaryotic proteins: tips for good practice as well as for bad. Cell Commun Signal.

[9] Krystkowiak I, Davey NE (2017). SLiMSearch: a framework for proteome-wide discovery and annotation of functional modules in intrinsically disordered regions. Nucleic Acids Res.

[10] Stoimenov I, Helleday T (2009). PCNA on the crossroad of cancer. Biochem Soc Trans.

[11] Gomes XV, Schmidt SL, Burgers PM (2001). ATP utilization by yeast replication factor C. II. Multiple stepwise ATP binding events are required to load proliferating cell nuclear antigen onto primed DNA. J Biol Chem.

[12] Majka J, Burgers PMJ (2004). The PCNA-RFC families of DNA clamps and clamp loaders. Prog Nucleic Acid Res Mol Biol.

[13] Fay PJ, Johanson KO, McHenry CS, Bambara RA (1981). Size classes of products synthesized processively by DNA polymerase III and DNA polymerase III holoenzyme of *Escherichia coli*. J Biol Chem.

[14] Tsurimoto T, Stillman B, Alberts BM (1990). Functions of replication factor C and proliferating-cell nuclear antigen: functional similarity of DNA polymerase accessory proteins from human cells and bacteriophage T4 (simian virus 40 DNA replication/DNA binding/ATPase/evolution). Biochemistry.

[15] Jarvis TC, Newport JW, Von Hippel PH (1991). Stimulation of the processivity of the DNA polymerase of bacteriophage T4 by the polymerase accessory proteins. The role of ATP hydrolysis. J Biol Chem.

[16] Reynolds N, Warbrick E, Fantes PA, Macneill SA (2000). Essential interaction between the fission yeast DNA polymerase δ subunit Cdc27 and Pcn1 (PCNA) mediated through a C-terminal p21(Cip1)-like PCNA binding motif. EMBO J.

[17] Dieckman LM, Washington MT (2013). PCNA trimer instability inhibits translesion synthesis by DNA polymerase η and by DNA polymerase δ. DNA Repair (Amst).

[18] Garg P, Burgers PMJ (2005). DNA polymerases that propagate the eukaryotic DNA replication fork. Crit Rev Biochem Mol Biol.

[19] Moldovan G-L, Pfander B, Jentsch S (2006). PCNA controls establishment of sister chromatid cohesion during S phase. Mol Cell.

[20] Gulbis JM, Kelman Z, Hurwitz J (1996). Structure of the C-terminal region of p21(WAF1/CIP1) complexed with human PCNA. Cell.

[21] Banks D, Wu M, Higa LA (2006). L2DTL/CDT2 and PCNA interact with p53 and regulate p53 polyubiquitination and protein stability through MDM2 and CUL4A/DDB1 complexes. Cell Cycle.

[22] Hasan S, Hassa PO, Imhof R, Hottiger MO (2001). Transcription coactivator p300 binds PCNA and may have a role in DNA repair synthesis. Nature.

[23] Abbas T, Sivaprasad U, Terai K (2008). PCNA-dependent regulation of p21 ubiquitylation and degradation via the CRL4Cdt2 ubiquitin ligase complex. Genes Dev.

[24] Centore RC, Yazinski SA, Tse A, Zou L (2012). Spartan/C1orf124, a reader of PCNA ubiquitylation and a regulator of UV-induced DNA damage response. Mol Cell.

[25] Moldovan G-L, Pfander B, Jentsch S (2007). PCNA, the maestro of the replication fork. Cell.

[26] Havens CG, Walter JC (2009). Docking of a specialized PIP Box onto chromatin-bound PCNA creates a degron for the ubiquitin ligase CRL4Cdt2. Mol Cell.

[27] Michishita M, Morimoto A, Ishii T (2011). Positively charged residues located downstream of PIP box, together with TD amino acids within PIP box, are important for CRL4(Cdt2) -mediated proteolysis. Genes Cells.

[28] Gilljam KM, Feyzi E, Aas PA (2009). Identification of a novel, widespread, and functionally important PCNA-binding motif. J Cell Biol.

[29] Olaisen C, Müller R, Nedal A, Otterlei M (2015). PCNA-interacting peptides reduce Akt phosphorylation and TLR-mediated cytokine secretion suggesting a role of PCNA in cellular signaling. Cell Signal.

[30] Boehm EM, Washington MT (2016). R.I.P. to the PIP: PCNA-binding motif no longer considered specific: PIP motifs and other related sequences are not distinct entities and can bind multiple proteins involved in genome maintenance. BioEssays.

[31] Bruning JB, Shamoo Y (2004). Structural and thermodynamic analysis of human PCNA with peptides derived from DNA polymerase-δ p66 subunit and flap endonuclease-1. Structure.

[32] Strzalka W, Oyama T, Tori K, Morikawa K (2009). Crystal structures of the *Arabidopsis thaliana* proliferating cell nuclear antigen 1 and 2 proteins complexed with the human p21 C-terminal segment. Protein Sci.

[33] Chapados BR, Hosfield DJ, Han S (2004). Structural basis for FEN-1 substrate specificity and PCNA-mediated activation in DNA replication and repair. Cell.

[34] Vijayakumar S, Chapados BR, Schmidt KH (2007). The C-terminal domain of yeast PCNA is required for physical and functional interactions with Cdc9 DNA ligase. Nucleic Acids Res.

[35] Matsumiya S, Ishino S, Ishino Y, Morikawa K (2002). Physical interaction between proliferating cell nuclear antigen and replication factor C from *Pyrococcus furiosus*. Genes Cells.

[36] Doré AS, Kilkenny ML, Jones SA (2006). Structure of an archaeal PCNA1-PCNA2-FEN1 complex: elucidating PCNA subunit and client enzyme specificity. Nucleic Acids Res.

[37] Xing G, Kirouac K, Shin YJ (2009). Structural insight into recruitment of translesion DNA polymerase Dpo4 to sliding clamp PCNA. Mol Microbiol.

[38] Duffy CM, Hilbert BJ, Kelch BA (2016). A disease-causing variant in PCNA disrupts a promiscuous protein binding site. J Mol Biol.

[39] Sakurai S, Kitano K, Yamaguchi H (2005). Structural basis for recruitment of human flap endonuclease 1 to PCNA. EMBO J.

[40] De Biasio A, de Opakua AI, Mortuza GB (2015). Structure of p15(PAF)-PCNA complex and implications for clamp sliding during DNA replication and repair. Nat Commun.

[41] Hoffmann S, Smedegaard S, Nakamura K (2016). TRAIP is a PCNA-binding ubiquitin ligase that protects genome stability after replication stress. J Cell Biol.

[42] Bubeck D, Reijns MAM, Graham SC (2011). PCNA directs type 2 RNase H activity on DNA replication and repair substrates. Nucleic Acids Res.

[43] Hishiki A, Hashimoto H, Hanafusa T (2009). Structural basis for novel interactions between human translesion synthesis polymerases and proliferating cell nuclear antigen. J Biol Chem.

[44] Wang Y, Xu M, Jiang T (2016). Crystal structure of human PCNA in complex with the PIP box of DVC1. Biochem Biophys Res Commun.

[45] Armstrong AA, Mohideen F, Lima CD (2012). Recognition of SUMO-modified PCNA requires tandem receptor motifs in Srs2. Nature.

[46] De Biasio A, Campos-Olivas R, Sánchez R (2012). Proliferating cell nuclear antigen (PCNA) interactions in solution studied by NMR. PLoS One.

[47] Hara K, Uchida M, Tagata R (2018). Structure of proliferating cell nuclear antigen (PCNA) bound to an APIM peptide reveals the universality of PCNA interaction. Acta Crystallogr Sect F Struct Biol Commun.

[48] Sebesta M, Cooper CDO, Ariza A (2017). Structural insights into the function of ZRANB3 in replication stress response. Nat Commun.

[49] Cino EA, Killoran RC, Karttunen M, Choy W-Y (2013). Binding of disordered proteins to a protein hub. Sci Rep.

[50] De Biasio A, Ibáñez De Opakua A, Cordeiro TN (2014). P15PAF Is an intrinsically disordered protein with nonrandom structural preferences at sites of interaction with other proteins. Biophys J.

[51] Yoon M-K, Venkatachalam V, Huang A (2009). Residual structure within the disordered C-terminal segment of p21(Waf1/Cip1/Sdi1) and its implications for molecular recognition. Protein Sci.

[52] Wegener KL, McGrath AE, Dixon NE (2018). Rational design of a 310-helical PIP-box mimetic targeting PCNA, the human sliding clamp. Chem Eur J.

[53] Salguero I, Guarino E, Shepherd MEA (2012). Ribonucleotide reductase activity is coupled to DNA synthesis via proliferating cell nuclear antigen. Curr Biol.

[54] Fleck O, Vejrup-Hansen R, Watson A (2013). Spd1 accumulation causes genome instability independently of ribonucleotide reductase activity but functions to protect the genome when deoxynucleotide pools are elevated. J Cell Sci.

[55] Vejrup-Hansen R, Fleck O, Landvad K (2014). Spd2 assists Spd1 in the modulation of ribonucleotide reductase architecture but does not regulate deoxynucleotide pools. J Cell Sci.

[56] Kjaergaard M, Brander S, Poulsen FM (2011). Random coil chemical shift for intrinsically disordered proteins: effects of temperature and pH. J Biomol NMR.

[57] Kjaergaard M, Poulsen FM (2011). Sequence correction of random coil chemical shifts: correlation between neighbor correction factors and changes in the Ramachandran distribution. J Biomol NMR.

[58] Zheleva DI, Zhelev NZ, Fischer PM (2000). A quantitative study of the in vitro binding of the C-terminal domain of p21 to PCNA: affinity, stoichiometry, and thermodynamics. Biochemistry.

[59] Kontopidis G, Wu S-Y, Zheleva DI (2005). Structural and biochemical studies of human proliferating cell nuclear antigen complexes provide a rationale for cyclin association and inhibitor design. Proc Natl Acad Sci USA.

[60] Nestoras K, Mohammed AH, Schreurs A-S (2010). Regulation of ribonucleotide reductase by Spd1 involves multiple mechanisms. Genes Dev.

[61] Shell SS, Putnam CD, Kolodner RD (2007). The N terminus of Saccharomyces cerevisiae Msh6 is an unstructured tether to PCNA. Mol Cell.

[62] Jones DT, Cozzetto D (2015). DISOPRED3: precise disordered region predictions with annotated protein-binding activity. Bioinformatics.

[63] Mészáros B, Erdős G, Dosztányi Z (2018). IUPred2A: context-dependent prediction of protein disorder as a function of redox state and protein binding. Nucleic Acids Res.

[64] Peng K, Radivojac P, Vucetic S (2006). Length-dependent prediction of protein intrinsic disorder. BMC Bioinform.

[65] Boomsma W, Frellsen J (2017) Spherical convolutions and their application in molecular modelling. In: 31st conference on neural information processing systems (NIPS 2017), Long Beach, CA, USA, pp 3436–3446

[66] Veselovsky AV, Ivanov YD, Ivanov AS (2002). Protein–protein interactions: mechanisms and modification by drugs. J Mol Recognit.

[67] Chen U, Chen S, Saha P, Dutta A (1996). p21Cip1/Waf1 disrupts the recruitment of human Fen1 by proliferating-cell nuclear antigen into the DNA replication complex. Proc Natl Acad Sci USA.

[68] Slade D (2018). Maneuvers on PCNA rings during DNA replication and repair. Genes (Basel).

[69] Fu D, Samson LD, Hübscher U, van Loon B (2015). The interaction between ALKBH2 DNA repair enzyme and PCNA is direct, mediated by the hydrophobic pocket of PCNA and perturbed in naturally-occurring ALKBH2 variants. DNA Repair (Amst).

[70] Stein A, Aloy P (2008). Contextual specificity in peptide-mediated protein interactions. PLoS One.

[71] Gorelik M, Davidson AR (2012). Distinct peptide binding specificities of Src homology 3 (SH3) protein domains can be determined by modulation of local energetics across the binding interface. J Biol Chem.

[72] Kelil A, Levy ED, Michnick SW (2016). Evolution of domain–peptide interactions to coadapt specificity and affinity to functional diversity. Proc Natl Acad Sci.

[73] Teyra J, Sidhu SS, Kim PM (2012). Elucidation of the binding preferences of peptide recognition modules: SH3 and PDZ domains. FEBS Lett.

[74] Palopoli N, González Foutel NS, Gibson TJ, Chemes LB (2018). Short linear motif core and flanking regions modulate retinoblastoma protein binding affinity and specificity. Protein Eng Des Sel.

[75] Rössig L, Jadidi AS, Urbich C (2001). Akt-dependent phosphorylation of p21(Cip1) regulates PCNA binding and proliferation of endothelial cells. Mol Cell Biol.

[76] Tsanov N, Kermi C, Coulombe P (2014). PIP degron proteins, substrates of CRL4Cdt2, and not PIP boxes, interfere with DNA polymerase g and i focus formation on UV damage. Nucleic Acids Res.

[77] Havens CG, Shobnam N, Guarino E (2012). Direct role for proliferating cell nuclear antigen in substrate recognition by the E3 ubiquitin ligase CRL4Cdt2. J Biol Chem.

[78] Kay L, Keifer P, Saarinen T (1992). Pure absorption gradient enhanced heteronuclear single quantum correlation spectroscopy with improved sensitivity. J Am Chem Soc.

[79] Wittekind M, Mueller L (1993). HNCACB, a high-sensitivity 3D NMR experiment to correlate amide-proton and nitrogen resonances with the alpha- and beta-carbon resonances in proteins. J Magn Reson Ser B.

[80] Grzesiek S, Bax A (1992). Correlating backbone amide and side chain resonances in larger proteins by multiple relayed triple resonance NMR. J Am Chem Soc.

[81] Kay LE, Ikura M, Tschudin R, Bax A (1990). Three-dimensional triple-resonance NMR spectroscopy of isotopically enriched proteins. J Magn Reson.

[82] Clubb RT, Thanabal V, Wagner G (1992). A constant-time three-dimensional triple-resonance pulse scheme to correlate intraresidue 1HN, 15N, and 13C′ chemical shifts in 15 N·13C-labelled proteins. J Magn Reson.

[83] Bax A, Davis DG (1985). MLEV-17-based two-dimensional homonuclear magnetization transfer spectroscopy. J Magn Reson.

[84] Bax A, Davis DG (1985). Practical aspects of two-dimensional transverse NOE spectroscopy. J Magn Reson.

[85] Derome AE, Williamson MP (1990). Rapid-pulsing artifacts in double-quantum-filtered COSY. J Magn Reson.

[86] Schleucher J, Schwendinger M, Sattler M (1994). A general enhancement scheme in heteronuclear multidimensional NMR employing pulsed field gradients. J Biomol NMR.

[87] Delaglio F, Grzesiek S, Vuister GW (1995). NMRPipe: a multidimensional spectral processing system based on UNIX pipes. J Biomol NMR.

[88] Kazimierczuk K, Orekhov VY (2011). Accelerated NMR spectroscopy by using compressed sensing. Angew Chem Int Ed Engl.

[89] Vranken WF, Boucher W, Stevens TJ (2005). The CCPN data model for NMR spectroscopy: development of a software pipeline. Proteins.

[90] Fiser A, Sali A (2003). ModLoop: automated modeling of loops in protein structures. Bioinformatics.

[91] Eisenberg D, Schwarz E, Komaromy M, Wall R (1984). Analysis of membrane and surface protein sequences with the hydrophobic moment plot. J Mol Biol.

[92] Jurrus E, Engel D, Star K (2018). Improvements to the APBS biomolecular solvation software suite. Protein Sci.

[93] Wagih O (2017). ggseqlogo: a versatile R package for drawing sequence logos. Bioinformatics.

[94] Holehouse AS, Das RK, Ahad JN (2017). CIDER: resources to analyze sequence-ensemble relationships of intrinsically disordered proteins. Biophys J.

