# Supplementary material for: The PCNA interaction motifs revisited: thinking outside the PIP-box
Source: Cell Mol Life Sci. 2019 May 27;76(24):4923–43. doi: 10.1007/s00018-019-03150-0 (PMC6881253; doi:10.1007/s00018-019-03150-0)
Supplement: Supplementary file 1 — Supplementary material 1 (DOCX 7507 kb) [file 18_2019_3150_MOESM1_ESM.docx]

SUPPLEMENTAL MATERIAL

Prestel, Wichmann, Martin et al


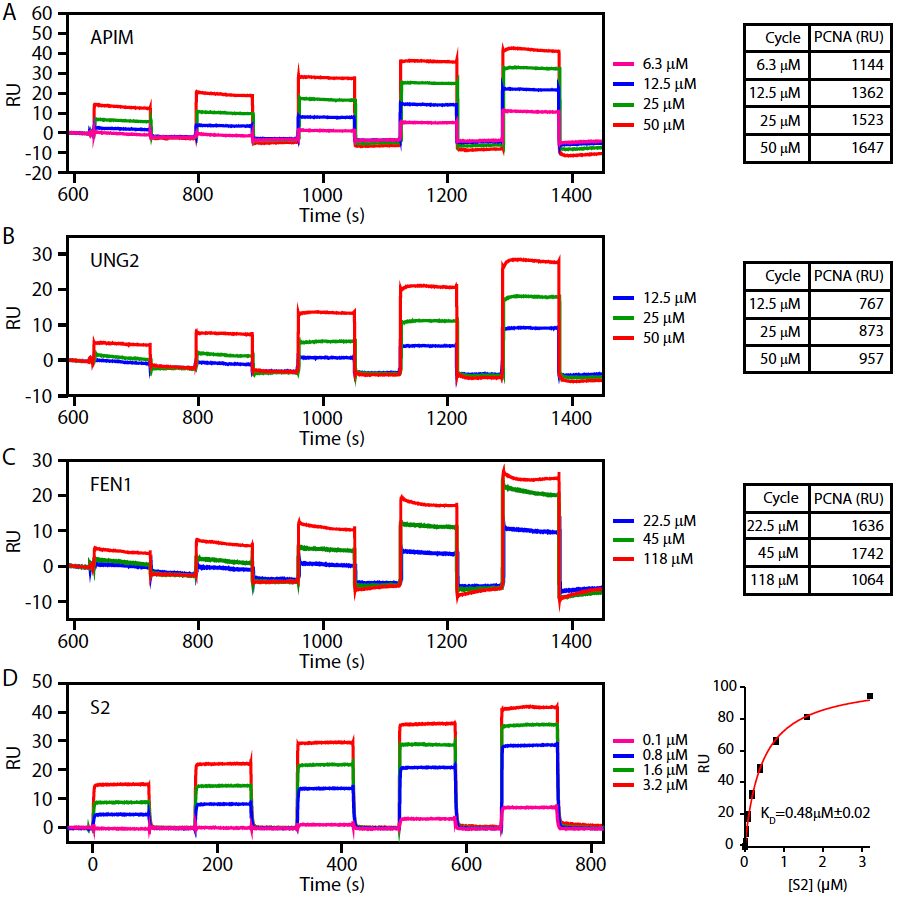


Fig. S1: SPR analyses of different peptide ligands binding to PCNA

The binding of A) APIM, B) UNG2, C) FEN1, and D) S2 to human PCNA measured by SPR. PCNA was captured using an immobilized Anti-His_6_ antibody. The sensorgrams were obtained by injecting five series of two-fold dilutions, injected in the order of increasing peptide concentration. The final concentration of each series is stated in the legend together with the capture level of PCNA of each injection cycle.

**
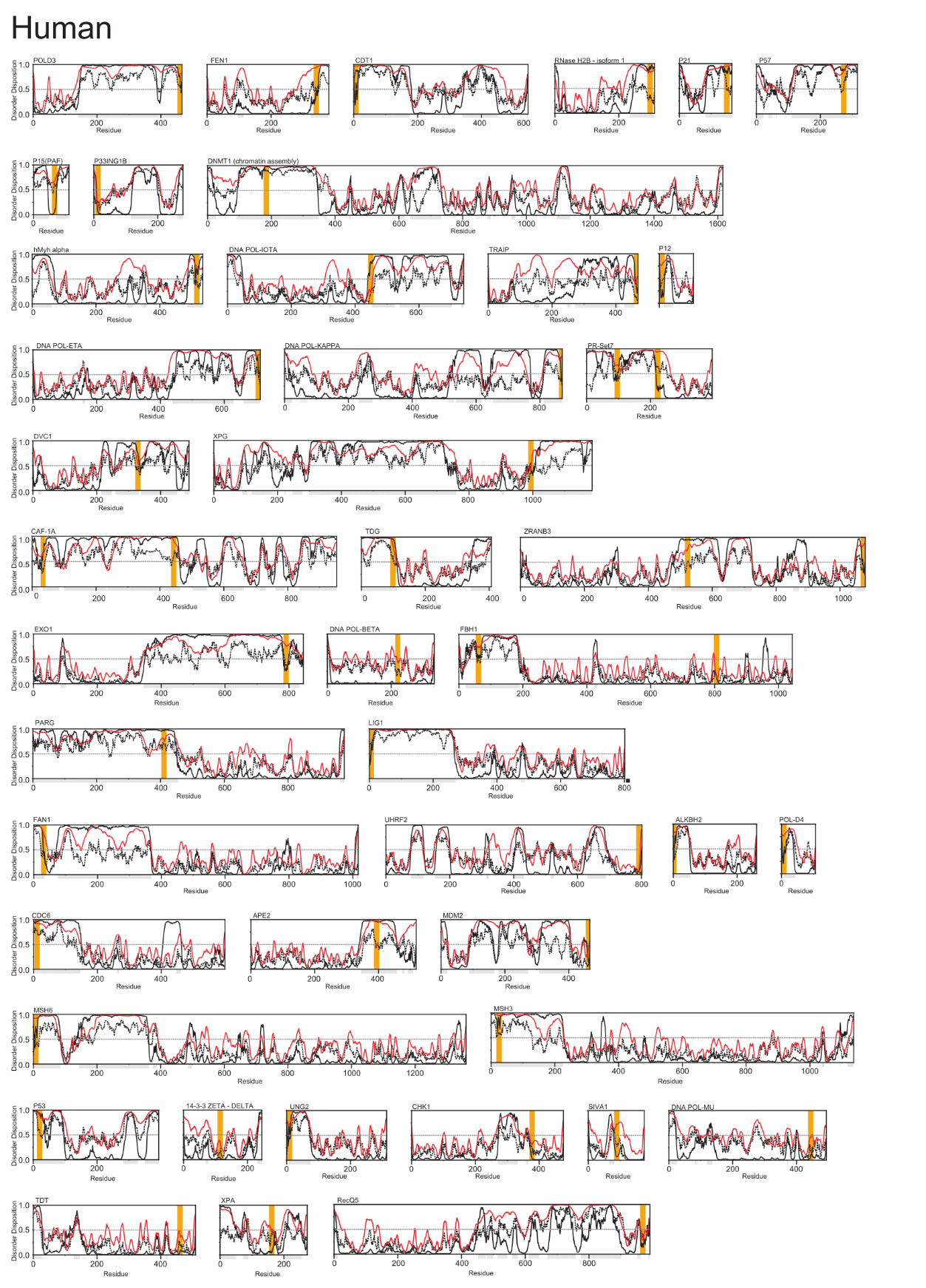
**

**
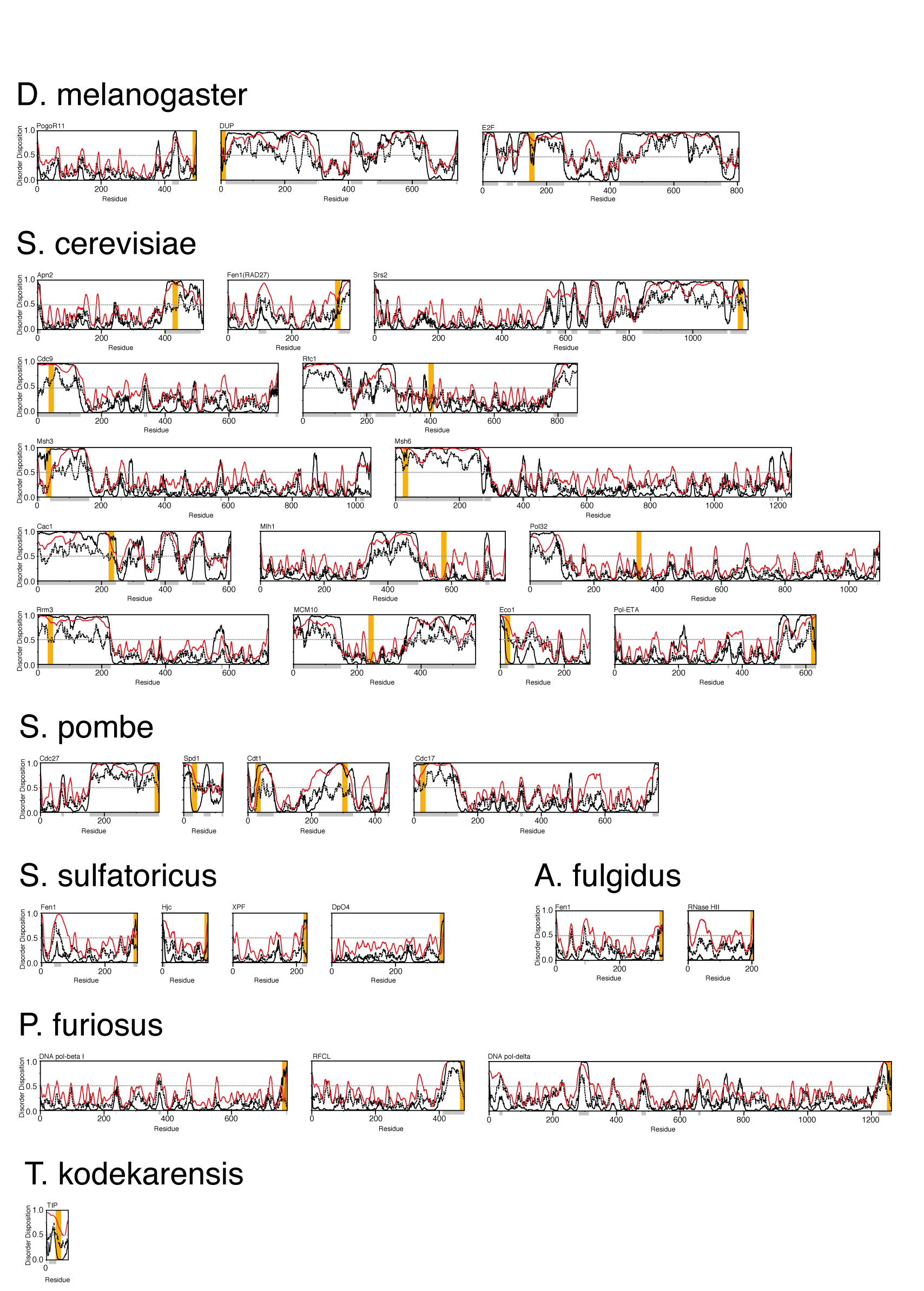
**

Fig. S2: Disorder predictions of PCNA ligands

The disorder propensity from 0 to 1 is plotted as a function of residue number and was predicted using Disopred3 (<http://bioinf.cs.ucl.ac.uk/psipred>) (black), IUpred2 (<https://iupred2a.elte.hu>) (striped black), and Pondr-fit VSL2 (<http://www.pondr.com>) (red) and default settings. The disorder for each residue was denoted with grey boxes below the x-axis by calculating the average disorder disposition for the three predictors with a threshold equal to or above 0.5 (indicated by grey dotted line). Orange boxes show PCNA binding motif-locations.


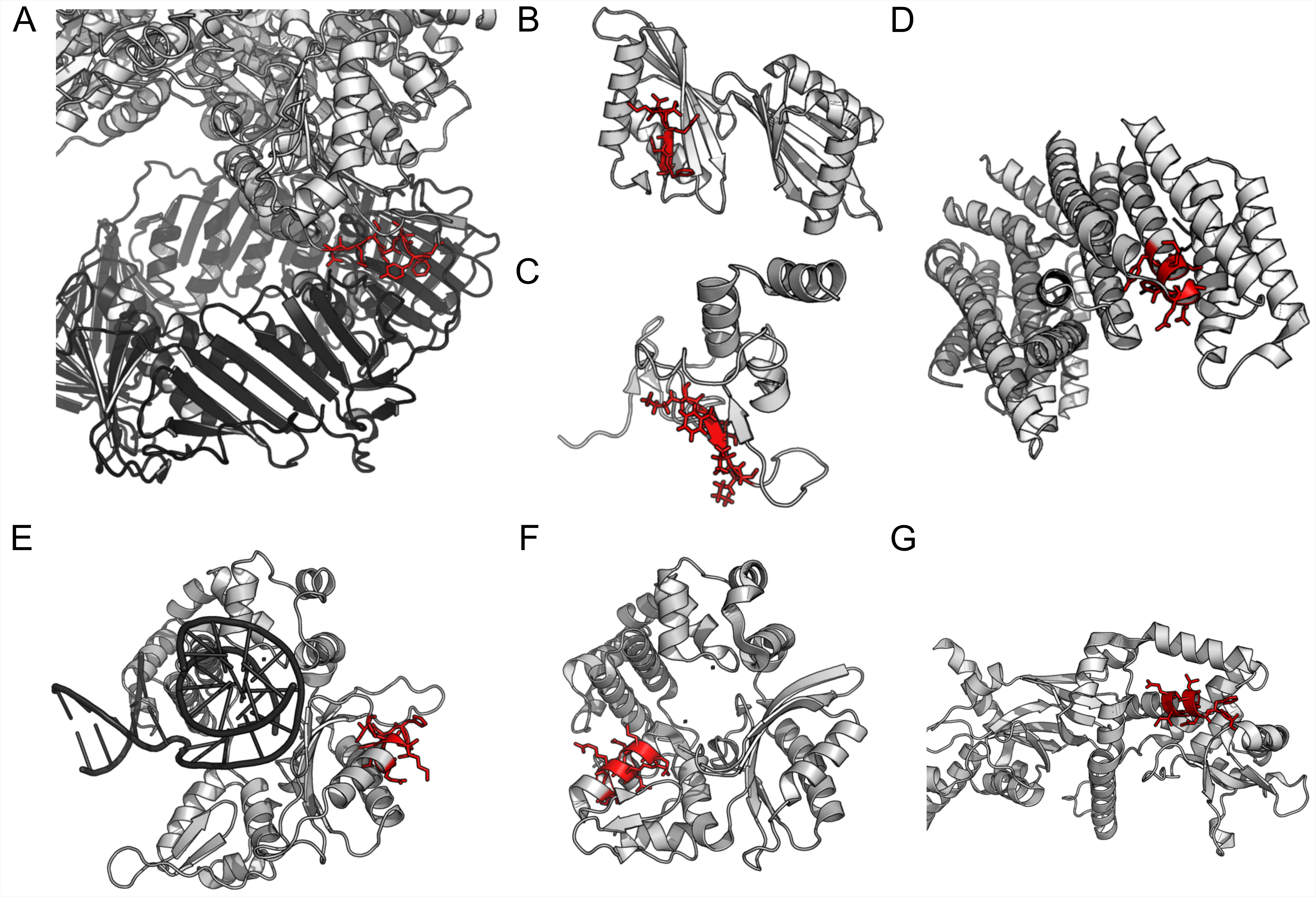


Fig. S3: PCNA binding motifs in folded domains.

A: Crystal structure of S. cerevisiae rfc1 in complex with PCNA (PDB-code 1SXJ (Bowman et al., 2004)) The sequence stretch that contains the PIP-motif (^398^**N**MS**V**VG**YF**^405^) is highlighted in red and the side chains are shown as sticks.

B: Crystal structure of human CHK1 (PDB-code 5WI2 (Emptage et al., 2017)). The sequence stretch that is suggested to contain the PIP-motif (^377^**M**TR**FF**^381^) is highlighted in red and the side chains are shown as sticks in one monomer of the dimeric structure.

C: Crystal structure of human XPA1 (PDB-code 1D4U (Buchko et al., 1999)). The sequence stretch that is suggested to contain the APIM-motif (^163^**KFIVK**^167^) is highlighted in red and the side chains are shown as sticks.

D: Crystal structure of human 14-3-3 ζ δ in complex with a phosphopeptide (PDB-code 1QJA (Gao et al., 2015)). The sequence stretch that is suggested to contain the PIP-motif (^111^**N**PE**S**KV**FY**^118^) is highlighted in red and the side chains are shown as sticks in one monomer of the dimeric structure.

E: Crystal structure of human DNA polymerase β in complex with DNA (PDB-code 1BPX). The sequence stretch that is suggested to contain the PIP-motif (^217^**Q**LQ**K**VH**FI**^224^) is highlighted in red and the side chains are shown as sticks.

F: Crystal structure of human DNA polymerase µ (PDB-code 4LZD). The sequence stretch that is suggested to contain the PIP-motif (^441^**Q**RE**L**RR**FS** ^448^) is highlighted in red and the side chains are shown as sticks.

G: Crystal structure of *S. cerevisiae* mlh1 in complex with pms1 (PDB-code 4E4W (Gueneau et al., 2013)). The sequence stretch that is suggested to contain the PIP-motif (^572^**Q**IG**L**TD**FA**^579^) is highlighted in red and the side chains are shown as sticks.


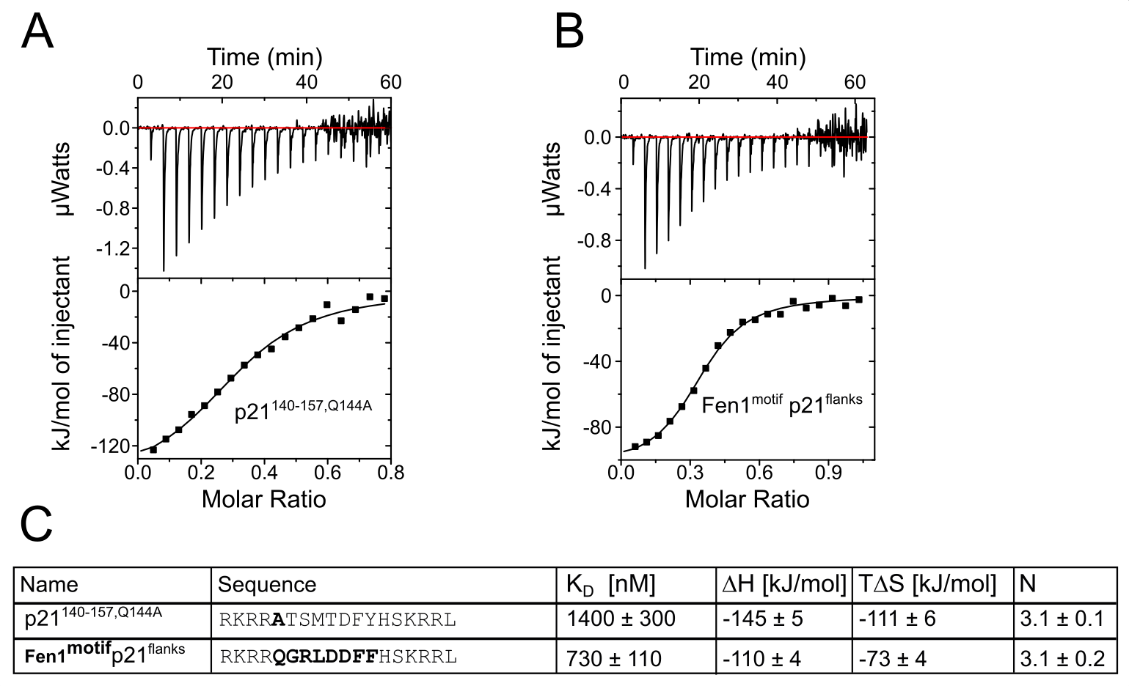


Fig. S4: ITC analyses of modified PCNA ligands.

A: Interaction of human PCNA with p21(Q->A) variant.

B: Interaction of human PCNA with p21-flanking regions grafted onto the motif of Fen1

C: Sequences of the used peptides and thermodynamic parameters of the interactions


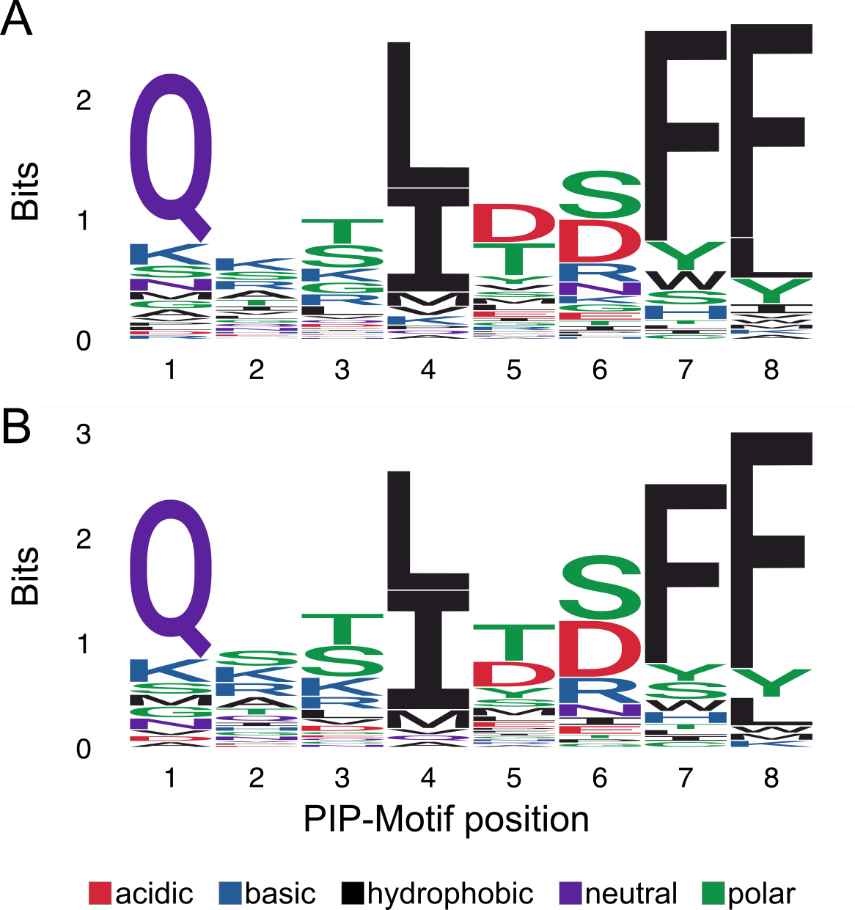


Fig. S5: Sequence LOGOs of the PCNA binding motifs from different classes.

A: Sequence logo of the amino acid distribution calculated from the motifs in Table 2 where binding has been determined by biophysical in vitro experiments (letter-height denotes information content).

B: Sequence logo of the amino acid distribution calculated from motifs in Table 2 which belongs to the classified disordered motifs (letter-height denotes information content).


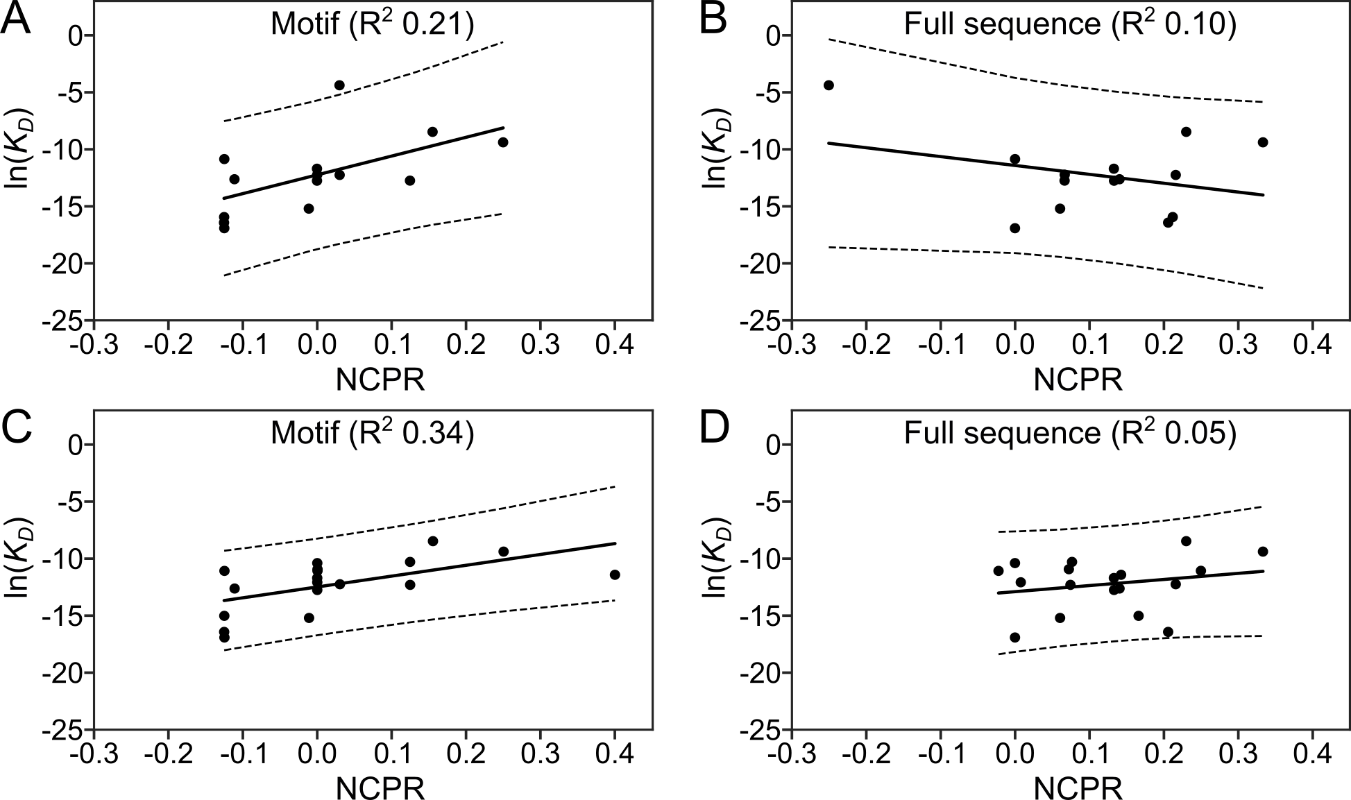


Fig. S6: Correlation variations of ln Kd versus NCPR.

A: Curated data set correlating *PIP motif* NCPR with experimental binding affinities for peptides longer than 15 residues. In cases where multiple affinities were available for the same protein, an average was used.

B: Curated data set correlating *full sequence* NCPR with experimental binding affinities for peptides longer than 15 residues. In cases where multiple affinities were available for the same protein, an average was used.

C: Curated data set correlating *PIP motif* NCPR with experimental binding affinities omitting the data for ING1. In cases where multiple affinities were available for the same protein, an average was used.

D: Curated data set correlating *full sequence* NCPR with experimental binding affinities omitting the data for ING1. In cases where multiple affinities were available for the same protein, an average was used.


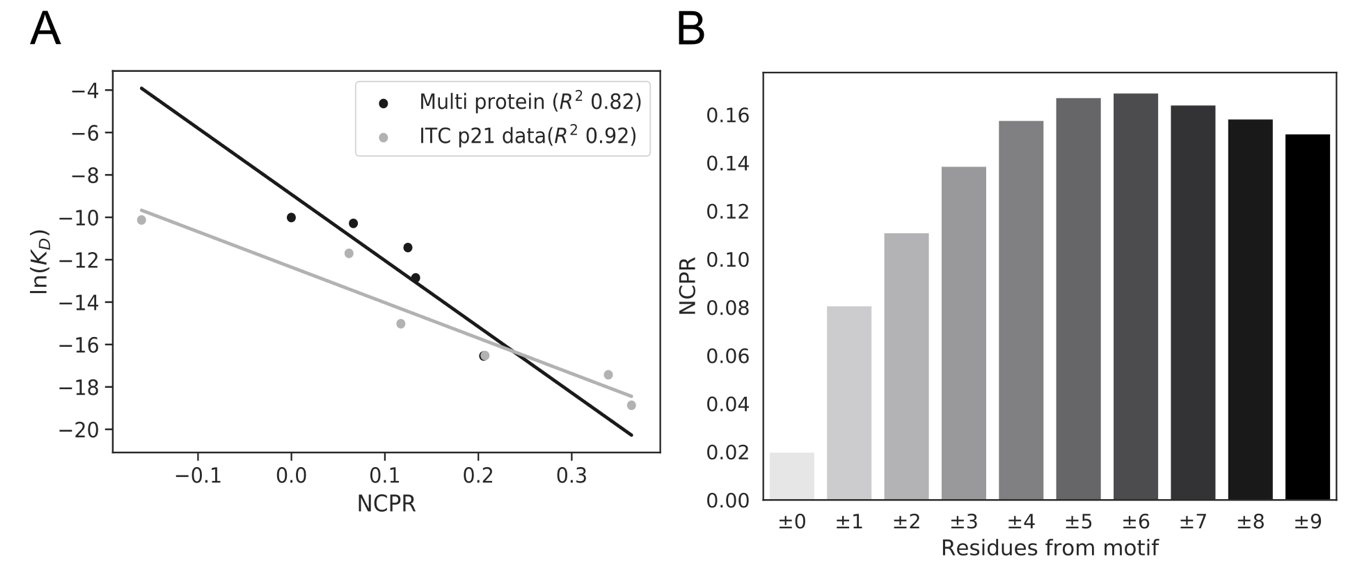


**Fig. S7.** Correlations between NCPR and various properties

A: Overlay of the correlation plots in Fig. 5B and Fig. 6F

B: Correlation between the length of the flanking regions and the NCPR for entries in Table 2 with a disorder propensity > 0.5

Table S1. Some identified PIP degrons

| **Species** | **Protein** | **Sequence of PIP motif(s)** | **PMID** | |
| --- | --- | --- | --- | --- |
| *H. Sapiens* | PR-SET7 | QQNRK**LTDFY**PVR**R**SS | 21035370 |  |
| *H. Sapiens* | TDG | EK**Q**EK**ITD**T**F**KVK**R**KV | 24962565 |  |
| *H. Sapiens* | p21 | RR**Q**TS**MTDFY**HSK**R**RL | 19595719 |  |
| *H. Sapiens* | FBH1 | GS**Q**RC**I**PE**FF**LAG**K**QP | 23677613 |  |
| *H. Sapiens* | CDT1 | ME**Q**RR**VTDFF**ARR**R**PG | 16407252 |  |
| *D. Melanogaster* | Dup | MA**Q**PS**V**AA**FF**TNR**K**RA | 20826610 |  |
| *S. pombe* | Cdt1 | GS**Q**TK**L**NFSVRKT**R**SS | 21493688 |  |
| *S. pombe* | Spd1 | SI**Q**GS**L**M**DV**GMRV**R**KS | 22464192 |  |
| *D. Melanogaster* | E2F | GKSND**IT**N**YY**KVK**R**RP | 19081076 |  |

Table S2: Binding affinities of curated PCNA binding proteins.

| Protein | Residues | | pH | T |  | K_D_ (M) | Sequence | PMID |
| --- | --- | --- | --- | --- | --- | --- | --- | --- |
| p21 | 139–160 | | 7.5 | 303 | ITC | 8.26E-08 | GRKRRQTSMTDFYHSKRRLIFS | 15576034 |
| p21 | 141–160 | | 7 | 303 | ITC | 5.26E-08 | KRRQTSMTDFYHSKRRLIFS | 10858286 |
| p21 | 141–160 | | 7 | 303 | ITC | 8.77E-08 | KRRQTSMTDFYHSKRRLIFS | 23139781 |
| p21 | 141–160 | | 7 | 308 | ITC | 1.00 E-07 | KRRQTSMTDFYHSKRRLIFS | 23139781 |
| p21 | 141–152 | | 7 | 308 | ITC | 1.10E-06 | KRRQTSMTDFYH | 23139781 |
| p21 | 141–152 | | 7 | 303 | ITC | 6.25E-07 | KRRQTSMTDFYH | 23139781 |
| p21 | 137–156 | | 7.5 | 298 | SPR | 6.50E-08 | SQGRKRRQTSMTDFYHSKRR | 19208623 |
| p21 | 143–157 | | 7.5 | 298 | ITC | 7.25E-08 | Ac-RQTSMTDFYHSKRRL-Am | This work |
| p21 | 143–157 | | 7.5 | 298 | SPR | 6.56E-08 | Ac-RQTSMTDFYHSKRRL-Am | This work |
| p12 | 1–19 | | 7.0 | 298 | ITC | 3.8E-07 | MGRKRKLITDSYPVVKRREGPAG | 30655288 |
| FEN1 | 332–353 | | 7.5 | 303 | ITC | 5.9E-05 | RQGSTQGRLDDFFKVTGSLSSA | 15576034 |
| FEN1 | 1–380 | | 7.4 | 298 | SPR | 6.00E-08 | MGIQGLAKLIADVAPSAIRENDIKSYFGRKVAIDASMSIYQFLIAVRQGGDVLQNEEGETTSHLMGMFYRTIRMMENGIKPVYVFDGKPPQLKSGELAKRSERRAEAEKQLQQAQAAGAEQEVEKFTKRLVKVTKQHNDECKHLLSLMGIPYLDAPSEAEASCAALVKAGKVYAAATEDMDCLTFGSPVLMRHLTASEAKKLPIQEFHLSRILQELGLNQEQFVDLCILLGSDYCESIRGIGPKRAVDLIQKHKSIEEIVRRLDPNKYPVPENWLHKEAHQLFLEPEVLDPESVELKWSEPNEEELIKFMCGEKQFSEERIRSGVKRLSKSRQGSTQGRLDDFFKVTGSLSSAKRKEPEPKGSTKKKAKTGAAGKFKRGK | 8876181 |
| FEN1 | 329–344 | | 7.4 | - | SPR | 8.46E-08 | SKSRQGSTQGRLDDFF | 24728180 |
| FEN1 | 1–380 | | 7.5 | - | SPR | 7.5E-08 | MGIQGLAKLIADVAPSAIRENDIKSYFGRKVAIDASMSIYQFLIAVRQGGDVLQNEEGETTSHLMGMFYRTIRMMENGIKPVYVFDGKPPQLKSGELAKRSERRAEAEKQLQQAQAAGAEQEVEKFTKRLVKVTKQHNDECKHLLSLMGIPYLDAPSEAEASCAALVKAGKVYAAATEDMDCLTFGSPVLMRHLTASEAKKLPIQEFHLSRILQELGLNQEQFVDLCILLGSDYCESIRGIGPKRAVDLIQKHKSIEEIVRRLDPNKYPVPENWLHKEAHQLFLEPEVLDPESVELKWSEPNEEELIKFMCGEKQFSEERIRSGVKRLSKSRQGSTQGRLDDFFKVTGSLSSAKRKEPEPKGSTKKKAKTGAAGKFKRGK | 10899134 |
| FEN1 | 336–350 | | 7.5 | 298 | SPR | 4.49E-05 | Ac-TQGRLDDFFKVTGSL-Am | This work |
| FEN1 | 329–350 | | - | 298 | ITC | 1.73∙10^-5^ | SKSRQGSTQGRLDDFFKVTGSL | 28621305 |
| APIM |  | | 7.5 | 298 | SPR | 1.09E-05 | Ac-MDRWLVKW-Am | This work |
| UNG2 | 1–15 | | 7.5 | 298 | SPR | 3.41E-05 | MIGQKTLYSFFTPSP | This work |
| MSH6 | 3–17 | | 7.5 | 298 | SPR | 2.61E-06 | Ac-RQSTLYSFFPKSPAL-Am | This work |
| TRAIP | 447–469 | | 7.4 | 298 | ITC | 3.07E-05 | KQRVRVKTVPSLFQAKLD TFLWS | 26711499 |
| p66Polδ | 451–466 | | 7.5 | 303 | ITC | 1.56E-05 | GKANRQVSITGFFQRK* | 15576034 |
| ING1 | 4–25 | | 7 | 308 | NMR | 1.25E-02 | PANGEQLHLVNYVEDTLDSIES | 23139781 |
| p15 | 1–111 | | 7 | 298 | ITC | 1.1E-07 | MVRTKADSVPGTYRKVVAARAPRKVLGSSTSATNSTSVSSRKAENKYAGGNPVCVRPTPKWQKGIGEFFRLSPKDSEKENQIPEEAGSSGLGKAKRKACPLQPDHTNDEKE* | 25762514 |
| p15 | 1–111 | | 7 | 308 | ITC | 2.5E-06 | MVRTKADSVPGTYRKVVAARAPRKVLGSSTSATNSTSVSSRKAENKYAGGNPVCVRPTPKWQKGIGEFFRLSPKDSEKENQIPEEAGSSGLGKAKRKACPLQPDHTNDEKE* | 25762514 |
| p15 | 32–111 | | 7 | 308 | ITC | 2.9E-06 | ATNSTSVSSRKAENKYAGGNPVCVRPTPKWQKGIGEFFRLSPKDSEKENQIPEEAGSSGLGKAKRKACPLQPDHTNDEKE | 25762514 |
| p15 | 50–77 | | 7 | 298 | ITC | 5.56E-06 | GNPVCVRPTPKWQKGIGEFFRLSPKDSE | 25762514 |
| p15 | 59–70 | | 7 | 308 | ITC | 8.3E-05 | PKWQKGIGEFFR | 25762514 |
| p15 | 50–77 | | 7 | 308 | ITC | 1.25E-05 | GNPVCVRPTPKWQKGIGEFFRLSPKDSE | 25762514 |
| PolEta | 696–713 | | 7.4 | 298 | ITC | 1.1E-05 | PRPEGMQTLESFFKPLTH* | 26903512 |
| PolEta | 694–713 | | 7.5 | 298 | SPR | 4.0E-07 | CKRPRPEGMQTLESFFKPLTH | 19208623 |
| FBH1 | 52–72 | | 7.5 | 279 | ITC | 2.5E-07 | RGQGSQRCIPEFFLAGKQPCTN | 23677613 |
| FBH1 | 605-620 | | 7.5 | 279 | ITC | 5.9E-07 | KDKFIRRWVHKEGFSG | 23677613 |
| ABH2 | 1-16 | | 7.5 | 279 | ITC | 3.2E-07 | MDRFLVKGAQGGLLRK | 23677613 |
| ZRANB3 | 511–532 | | 7.5 | 298 |  | 4.8E-06 | FTHFEKEKQHDIRSFFVPQPKK | 28621305 |
| ZRANB3 | 1058–79 | |  | 298 | ITC | 9.2E-06 | QVRRQSLASKHGSDITRFLVKK | 28621305 |
| PolIota | 438–459 | |  | 298 | ITC | 5.5E-06 | LKALNTAKKGLIDYYLMPSLST | 28621305 |
| PolIota | 419–434 | | 7.5 | 298 | SPR | 3.9E-07 | CAK**K**GL**I**DY**YL**MPSLST | 19208623 |
| PolIota | 444–454 | | 7.4 | 298 | ITC | 7.8E-06 | AKKGLIDYYLM | 26903512 |
| Exo | 783–804 | | 7.5 | 303 | ITC | 8.3E-06 | NKPGLQIKLNELWKNFGFKKDS | 20970388 |
| PARG | 402–420 | | 7.4 | 298 | ITC | 3.3E-06 | QHGKKDSKITDHFMRLPKA | 28934471 |
| p12 | 1-19 | | 7.0 | 298 | ITC | 3.8E-05 | MGRKRLITDSYPVVKRREG | 30655288 |
| p12 | 1-19 | | 7.0 | 308 | NMR | 1.3E-04 | MGRKRLITDSYPVVKRREG | 30655288 |
| RecQ5 | 952-979 | | 7.0 | 308 | ITC | 2.1E-04 | KTSPGRSVKEEAQNLIRHFFHGRARCES | 30655288 |
|  | |  | | | | | | |
